# Using archaeological and geomorphological evidence for the establishment of a relative chronology and evolution pattern for Holocene landslides

**DOI:** 10.1371/journal.pone.0227335

**Published:** 2019-12-31

**Authors:** Mihai Niculiţă, Mihai Ciprian Mărgărint, Alexandru Ionuţ Cristea

**Affiliations:** 1 Department of Geography, Geography and Geology Faculty, Alexandru Ioan Cuza University of Iaşi, Iaşi, Romania; 2 Department of Geography, Ştefan cel Mare University, Suceava, Romania; University at Buffalo - The State University of New York, UNITED STATES

## Abstract

Hilly regions around the world are one of the most vulnerable places for inhabitation, where landslides represent a permanent threat for their population. In some particular cases, in the past, due to their topographic features, areas affected by massive landslides served a real opportunity for the location of strategic and fortified settlements. In this study, we have extended a previous approach of correlation between landslides and archaeological heritage, adding 14 new representative case studies of landslided hillforts, a new period with landslided hillforts, and a new typology of relationship (landslided tumuli) for establishing relative chronologies for landslide inventories. The landslide mapping presented here supplements a previous inventory, which now has 1211 landslides, and it is based on the interpretation of high-resolution DEMs, geomorphometric derivatives, remote sensing images, and field validation. For one of the most characteristic sites (Băiceni settlement, Iaşi County), we used Electrical Resistivity Tomography (ERT) to assess the geometry of the compound and complex landslides. The current approach allowed us to acquire a more accurate relative chronology of landslide activity during the Holocene and Upper Pleistocene, and more importantly, to establish the pattern of landslides evolution in the Moldavian Plateau, North-Eastern Romania. The relict landslides are Lateglacial and Lower Holocene, the old landslides are post-Holocene Climatic Optimum and pre-Medieval, while the recent landslides are post-Medieval. The landslide magnitude decreased continuously, the new events being retrogressive reactivations of earlier events scarps and landslide bodies (as shown by the ERT data). Further studies on absolute dating will improve the relative chronology. Still, while not all the landslides can be dated, the methodology that we describe can be applied to increase the spatial density of the relative chronology. The presented approach can be used in other regions all over the world to establish the relative age of landslide inventories when archaeological topography can be related to landslide topography.

## Introduction

Landslides are geomorphological, hydrological, and geological processes that shape the Earth’s surface in various climatic conditions and contexts [1–3]. The resulted landforms and deposits may remain as evidence of past landslides, from the Holocene [4,5], the Pleistocene [6,7] or even earlier periods [8], particularly in dry climates [9]. For certain areas, there is evidence of a local continuous spatial and temporal clustering of landslides all over the Upper Pleistocene and Holocene [10–13], although it seems that toward nowadays globally, the landslide magnitude is decreasing [14].

Landslides are important agents of landform evolution [15–20] and lead to ecologic and environmental changes [21,22]. In addition to this, landslides acted and still act as natural hazards [23], posing risks and generating human and economic losses to society [24–28] and becoming a global problem [1]. Besides earthquakes and volcanic activity, landslides are the leading cause of deaths at global scale [14,29].

The identification and the mapping of anthropic and natural landforms have increased in the recent years through photo-interpretation of high-resolution LiDAR DEM’s [30–35]. Landslide research shows signs of progress in the field of landslides’ relative age and typology determination [30,36]. The subsurface geometry of landforms can be more accurately identified through a combined interpretation of LiDAR DEM’s with Electrical Resistivity Tomography (ERT) profiles [37,38].

**Fig 1:**
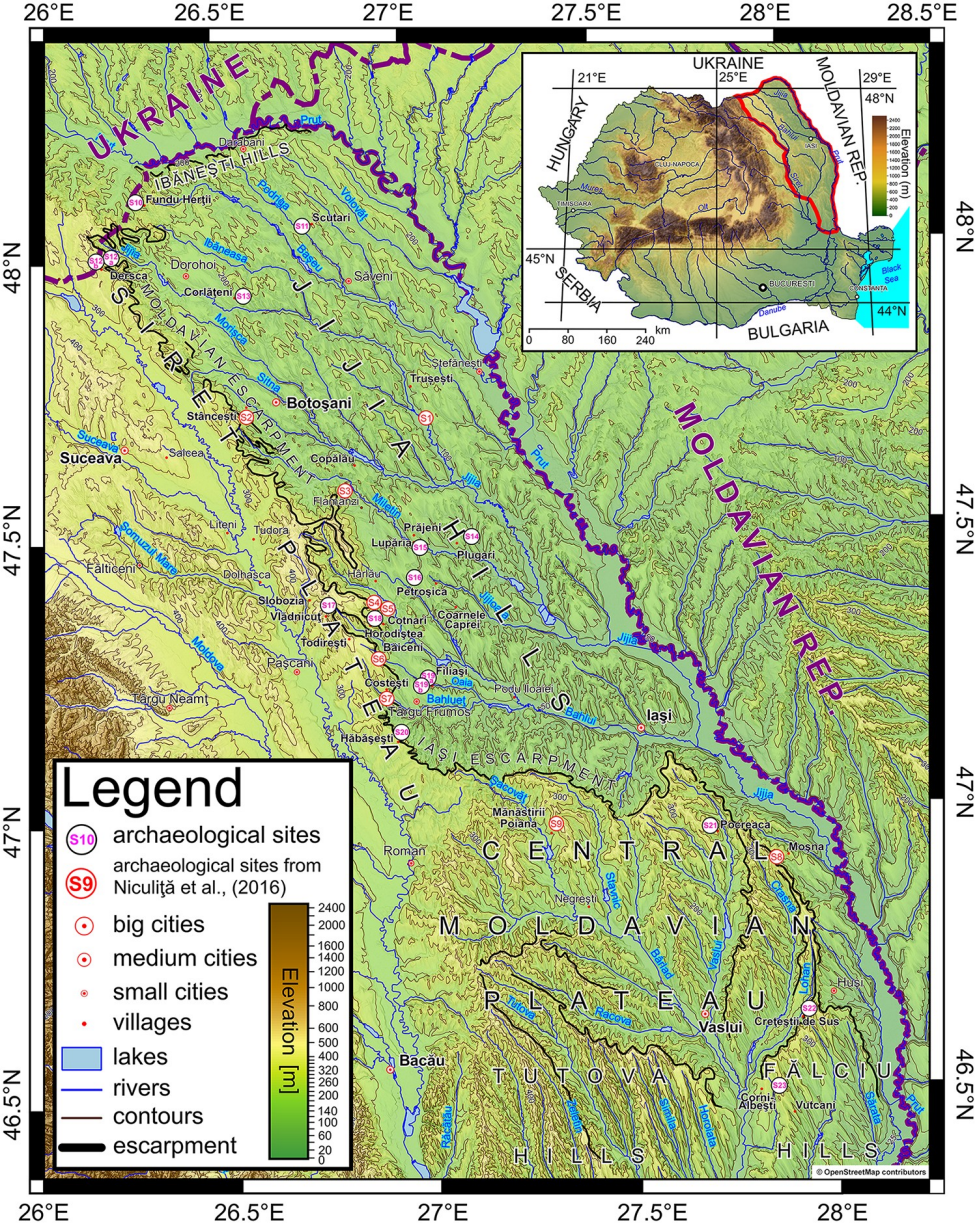
The geographic position of the studied sites in the Central and Northern part of the Moldavian Plateau: S1—9 are described in [39], S10—Fundu Herţii—La Redută, S11— Scutari—La Gheţărie, S12a— Dersca—La Pisc, S12b— Dersca—Berezna, S13— Corlăţeni—Movila Cetăţii, S14— Plugari—Movila Balş, S15— Prăjeni—Movila Robului, S16— Coarnele Caprei—Movila Boului, S17— Todireşti—La Şanţuri, S18—Cotnari—Horodiştea, S19a—Filiaşi—Dealul Mare-Boghiului, S19b—Filiași—Movila Boghiului, S20—Hăbăşeşti—La Silişte, S21—Pocreaca—Punct Cetăţuia, S22—Creţeşti—Dealul Cetăţii, S23—Vladnic (the background layer is a hypsometric map derived from SRTM 1 Arc-Second Global, https://doi.org/10.5066/F7PR7TFT). https://doi.org/10.6084/m9.figshare.11320127.v1.

The present work is an extended chronology and evolution pattern of the Holocene landslide activity from the Moldavian Plateau, Romania, completing the [39] database, both spatially and temporally, with a number of 14 new sites (Fig 1) and corresponding landslide inventories, emphasizing: (i) the medieval period (the period between the VIIIth to XIth centuries) with landslides activity and hillforts which provide a temporal reference regarding the relative age of landslides, (ii) more case-studies of the pre-Cucutenian landslides, (iii) more case-studies of the Hallstatt hillforts affected by landslides, (iv) a new type of relationship between archaeological remains and landslides—Bronze Age tumuli on landslided landforms which provide also another valuable temporal reference regarding the relative age of landslides. All the mapped landslides from this present work and those previously mapped by [39] were analyzed using the landslide area distributions for each of the relative landslide ages (relict, old and recent) to assess the completeness of the inventories. Based on the findings regarding the relative age and the Electrical Resistivity Tomography (ERT) profile from the representative Băiceni complex landslide (Iaşi County), we established the geometry of the landslides and also were able to provide a conceptual evolution pattern of these landslides.

## Study area

### The geological and geomorphological framework of landslide activity

An extensive description of the geology, geomorphology, and of the general features of the landslides from the Moldavian Plateau can be found in [39–43]. In this study, we will emphasize mainly the relations between lithology and landslides.

The caprock geological structure with escarpments and cuestas broadly defines the landforms of the Moldavian Plateau [40,42] and strongly influences the pattern of landslide distribution and activity [42,44].

In the northern part of the Moldavian Plateau, the Prut River incision and homoclinic shifting toward south generated a 30 km long cuesta escarpment (with up to 150 m relative altitude) exposing the caprock structure on its right valley side [45]. The Prut River is incised in a geological suite that starts with Cretaceous (Cenomanian) limestones followed by transgressive Badenian conglomerates with flint (which laterally become sands with a 30–40 m thickness), gypsum (60 m thick), limestones and marls with a 20 m thickness [46]. The Volhynian rocks transgressively covered the Badenian suite and were split stratigraphically in a lower member (150 m thick) and an upper member (100 m thick) [46–48]. The lower member presents an alternation of sands, quartz arenites, oolitic calcarenites, mudstones (Bajura Clay Formation), and andesitic tuffs (Hudeşti tuff, 2–5 m thick) [46]. The upper member presents an alternation of claystones, siltstones, mudstones, sands, quartz arenites and oolitic calcarenites, with bentonitic intercalations—the Dărăbani-Mitoc Clay Formation [47]. The Jijia and Prut rivers, tributaries are incised and generated a fragmented hilly area with an altitude of the ridges ranging between 250 and 300 m a.s.l.—the Ibăneşti Hills (Fig 1).

These strata dip towards south being covered by Bessarabian rocks (south of the alignment made by the Ştefăneşti-Truşeşti-Copălău-Tudora localities). The altitude of the ridges in this hilly area–the Jijia Hills–is generally under 225 m a.s.l. In this region, the escarpment structures are not developed because the limestones layers are very thin [49]. Nonetheless, cuesta landforms are occurring due to the river homoclinic shifting and to the alternation of mudstones, siltstones, claystones and sand layers. On the steep scarp slopes, the bedrock is close to the surface, and bedrock traces can be followed along the entire hillslopes (they influence the hillslope morphology and appear as steps in a transversal section of the hillslope), especially in the areas dominated by soil erosion and landslides.

In the central part of the study region, this lithological pattern and the Bahlui River homoclinic shifting towards south generated one of the most impressive escarpments of the Moldavian Plateau, the Iaşi Escarpment (Fig 1) [42]. This escarpment stretches between the Siret and Prut river valleys, on a west to east direction over more than 60 km in length, with relief ranging up to 350 m. The Bessarabian rocks (around 400 m thick) start with a succession of marls, siltstones, mudstones and claystones with sandy intercalations and present two facies: the eastern facies (toward the Prut Valley) is dominantly argillaceous, while the western one (toward the Siret Valley) is littoral-neritic with more sand and sandstone intercalations [50]. At the upper part of the Bessarabian, the Repedea Limestone Formation (calcarenites and oolitic calcarenites) and sandstones (quartz arenites), 10 to 25 m thick, overlays a sandy formation (Bânova-Muntele Formation—230 m thick) and siltstones, claystones and mudstones with centi- and decametric intercalation of sands (Cryptomactra Clay Formation– 200 to 300 m thick) [47,50,51]. Toward south and east, Repedea Formation has lateral facies variation characterized by the increase of the sand and sandstone content [50]. The Bessarabian formations are overlaid by Kersonian rocks, predominantly composed by sands and sandstones with clayey intercalations, 100 m thick [50].

In the hilly area east of Siret River (the Siret Plateau [52]), in the lower Bessarabian, over the Volhynian rocks, a lateral facies occurs, being characterized by the presence of a level of sands with gravels (40–50 m thick), followed by three levels of oolitic calcarenites, 0.5 to 5 m thick at 5 m vertical distance: the lower Hărmăneşti level, the upper Hărmăneşti level and the Crivești level [53]. This caprock structure generated the massiveness of this hilly area, where altitudes of the ridges go up to 586 m a.s.l. (Tudora-Dealu Mare). The extension toward the west of the Jijia and Bahlui tributaries and the caprock geology generated the so-called Moldavian Escarpment [52,54], which is more than 80 km long and has a relief that can reach 300–450 m a.s.l. These escarpments are affected by complex landslides, as in the case of the Băiceni archaeological site [55].

In the eastern part of the Central Moldavian Plateau, Tutova Hills and Fălciu Hills the caprock escarpments are given by the presence of the Meotian cineritic Nuţaşca-Ruseni Formation (10–20 m thick in the east, near the Prut valley and 40–60 m thick in the west, near the Siret valley) [56,57] over the Kersonian sands and clays intercalations (130–150 m thick) [58]. This cineritic formation has three layers of andezitic cinerites interlayered by clays and sands, which dip toward south-east [59]. Through the incision and homoclinic shifting the most important rivers from this area (Racova, Crasna, Lohan, and Vaslui) have created cuesta escarpments where landslides develop intensively [42,60].

For the last several hundreds of years, landslide triggering was related to rainfall multiannual trends and snowmelt [61]. The earthquake triggered landslides events [62,63] cannot be excluded, but for the moment, we are not able to relate any mapped landslide to past earthquakes.

Spatial and temporal patterns of landslide distribution similar to our study region can be found in many monoclinic regions of Europe [64–66]. Certain specific characteristics of these examples will be detailed in the discussion section of this paper.

### Climate and paleogeography of Late Pleistocene and Holocene Moldavian Plateau

The general characteristics of the Last Glacial Maximum, Lateglacial and Holocene for the Central and Eastern Europe are used to draw some ideas about the environment of North-Eastern Romania because to the present day there are no detailed reconstructions of the paleo-environment in the Moldavian Plateau. There are only two exceptions with regard to the fluvial environments of the Siret River which were reconstructed by [67] for the last 6000 years based on wood C14 chronology, and another one regarding the fluvial and lake sediments of Romania, reconstructed by [68], for the last 12000 years using probability density functions (PDFs) of radiocarbon data.

Wet periods (also shown by the tree large radial growth), when hydro-geomorphological activity was higher, were identified [68] in the following periods: 6600–5700, 3700–2900, 2300–1900, 1000–900, 750–650, 450–350, 150–50 yr. BP. Spikes in humidity shown by tree ring growth for the last 12000 years are 10300, 9800, 9200, 8500, 8000, 6790, 5650, 3750, 3300, 2850, 2350, 1500, 1300, 880 and 500, while for the last 200 years these are 1824–1825, 1870–1871, 1890–1991, 1926, 1970 and 1997. Between these periods, the climate was dryer (as shown by the tree small radial growth), especially between 3200–3150, 2775–2700, 1400 yr. BP [67].

In the vicinity of our study area, detailed paleoclimatic reconstructions were made at the Bukovinka Cave, at the foothill of the Ukrainian Carpathians [69,70]. The cave deposits begin with fluvial layers covered by a thick deposit dated to 10 730±60 cal yr BP and cover the period up to 700–200 yr BP. The vegetation and animal fossils allowed the identification of the Allerød interstadial or an older one, the Young Dryas, and all the Holocene subdivisions, including the Little Ice age. The Late Glacial period was dominated by a wet and cold temperate climate with frequent soil disturbances and loess particle deposition (conclusions of [69] based on pollen analysis).

Young Holocene deposits are eroded, the mid Holocene sandy deposits, having fluvial origin, and the pollen indicate a warm and humid period with broad-leaved forests (*Carpinus betulus*, *Querqus robur*, *Ulmus sp*., *Fraxinus sp*., *Fagus sylvatica*, *Tilia cordata*, T. platyphyllos, *Cornus sp*., *Abies sp*., *Alnus sp*., *Ericaceae*, *Polypodiaceae*) which can be associated with the Mid Holocene climatic optimum [69].

At the end of the Atlantic and in the Subboreal a layer with large silt particles, coprolites, and fragments of bones of *Marmota bobak* show an increase in the aridity with an open steppe landscape [69]. Subboreal sediments were identified using the presence of the magnetic signature of the 2.8 ka BP excursion of the Ukrainian Holocene magnetostratigraphy framework [69,70]. The pollen analysis showed a humid climate with coniferous and broad-leaved forests (*Picea*, *Abies*, *Carpinus*, *Querqus*, *Tilia*, *Corylus*), while the presence of *Cerealia* pollen indicates the correspondence with the agriculture of the Late Bronze Age [69]. Early Atlantic was wet and cold (*Abies*, *Picea*, *Pinus cembra*, *Alnus*, *Fagus*, *Carpinus*, *Cyperaceae*) and followed by an arid and warm climate, (the Middle Subatlantic), pollen data showing an increase in broad-leaved and bushes species [69].

In the upper part, of this suite, a light-colored loam with coarse silt is associated with the Little Ice Age (700–200 y BP), based on the decrease of wet-loving plants and an increase in NAP (Non Arboreal Pollen)—*Chenopodiaceae*, *Asteraceae* and *Cichoriaceae*, followed by an increase in *Pinus* pollen, corresponding to the post-medieval forest clearance [69].

### The habitation of the Moldavian Plateau and the study sites

A description of the cultures from the Neolithic, Bronze Age, Iron Age, and the medieval period from the study area is provided in [39]. The selected 14 archaeological sites (Fig 1) are mainly located on hilltops, either as fortified settlements or as burial mounds (Table 1). A detailed description of the site’s archaeology and geomorphology is given in the S1 Annex.

**Table 1:** List of the archaeological sites examined in this work, and their archaeological situation (S = settlement, F = fortress, FS = fortified settlement, T = tumuli); first and last cultures names and codes refer to Table 1 from [39]; S10 to S23 are the site numbers; for their geographic locations see Fig 1.

**Archaeological sites**				
**Id**	**S10**	**S11**	**S12a**	**S12b**
**Label**	Fundu Herţii–La Redută	Scutari–La Gheţărie	Dersca–La Pisc	Dersca—Berezna
**Official name**	Situl arheologic de la Fundu Herţii—La Redută	Aşezarea Cucuteni de la Scutari—La Gheţărie	Aşezarea fortificată medievală de la Dersca-La Pisc	Aşezarea Hallstatt de la Dersca—Berezna
LMI codek*	BT-I-s-B-01786	-	-	BT-I-s-A-01777-
RAN code**	36998.01	38358.01	37020.02	37020.01
**Latitude**	48° 06' 17.6025" N	48° 03' 26.6362" N	48° 00' 25.7163" N	48° 00' 27.5675" N
**Longitude**	26° 18' 39.8564" E	26° 44' 56.3474" E	26° 13' 28.2898" E	26° 12' 52.4918" E
**County**	Botoșani	Botoșani	Botoșani	Botoșani
**Type**	F	S	F	F
**Cultures**	CAC, VIII-IX, IX-XI	CAC, CBC	IX-XI	HA
**Main references**	[71]	[72–74]	[71]	[75]
**Archaeological sites**				
**Id**	**S13**	**S14**	**S15**	**S16**
**Label**	Corlăţeni–Movila Cetăţii	Plugari–Movila Balş	Prăjeni–Movila Robului	Coarnele Caprei–Movila Boului
**Official name**	Movila din Dealul Cetăţii de la Corlăteni	Tumulii de la Plugari	Movila din Valea Robului de la Lupăria	- -
LMI code*	-	IS-I-s-B-03634	-	- -
RAN code**	36685.16	98346.03	38615.14	-
**Latitude**	47° 56' 08.8795" N	47° 30' 02.6874" N	47° 29' 01.3367" N	47° 25' 50.7263" N
**Longitude**	26° 34' 49.7295" E	27° 09' 49.6284" E	27° 01' 41.9876" E	27° 00' 42.4224" E
**County**	Botoșani	Iași	Botoșani	Iaşi
**Type**	T	T	T	T
**Cultures**	HEC; JC	JC	JC	JC
**Main references**	[76,77]	http://ran.cimec.ro/sel.asp?codran=98346.03	http://ran.cimec.ro/sel.asp?codran=38615.14	
**Archaeological sites**				
**Id**	**S17**	**S18**	**S19a**	**S19b**
**Label**	Todireşti–La Şanţuri	Cotnari—Horodiştea	Filiaşi–Dealul Mare-Boghiului	Filiaşi–Movila Boghiului
**Official name**	Situl arheologic de la Todireşti- La Şanţuri	-	Aşezarea Cucuteni de la Filiaşi—Dealul Mare	Movila Boghiului
LMI code*	IS-I-s-B-03669	-	-	- -
RAN code**	99548.01	-	95827.01	-
**Latitude**	47° 23' 06.3267" N	47° 21' 32.1135" N	47° 15' 08.4692" N	47° 14' 36.3109" N
**Longitude**	26° 47' 10.2682" E	26° 54' 21.6919" E	27° 02' 27.2835" E	27° 02' 40.2154" E
**County**	Iași	Iași	Iași	Iași
**Type**	F	F	FS, T	T
**Cultures**	CAC, LTA	HD-LTC	CAC	JC
**Main references**	*http*:*//ran*.*cimec*.*ro/sel*.*asp*?*codran = 99548*.*01*	[78,79]	[80]	-
**Archaeological sites**				
**Id**	**S20**	**S21**	**S22**	**S23**
**Label**	Hăbăşeşti–La Silişte	Pocreaca–Punct Cetăţuia	Creţeşti–Dealul Cetăţii	Corni-Albeşti–Vladnic
**Official name**	Situl arheologic de la Hăbăşeşti—La Silişte	Situl arheologic de la Schitu Duca-Punct Cetăţuia	Cetatea de pământ Latene de la Creţeşti—Dealul Cetăţii	Cetatea Latene de la Corni-Albeşti
LMI code*	IS-I-m-A-03597.01	-	VS-I-s-B-06665	-
RAN code**	99281.01	98863.01	163217.02	162032.01
**Latitude**	47° 09' 26.4335" N	46° 58' 33.3439" N	46° 38' 45.4425" N	46° 30' 40.7758" N
**Longitude**	26° 58' 05.5236" E	27° 45' 34.4644" E	27° 59' 48.6892" E	27° 54' 47.6342" E
**County**	Iași	Iași	Vaslui	Vaslui
**Type**	FS	F	**F**	**F**
**Cultures**	CAC, LTB, IV, XV-XVI, XVII-XVIII	CAC, HB	LTA	LTA
**Main references**	[81	[82]	[83]	

* Historic monument list,

**National Archaeological Repertoire (http://ran.cimec.ro/)

## Material and methods

### Landslide inventories

**Fig 2:**
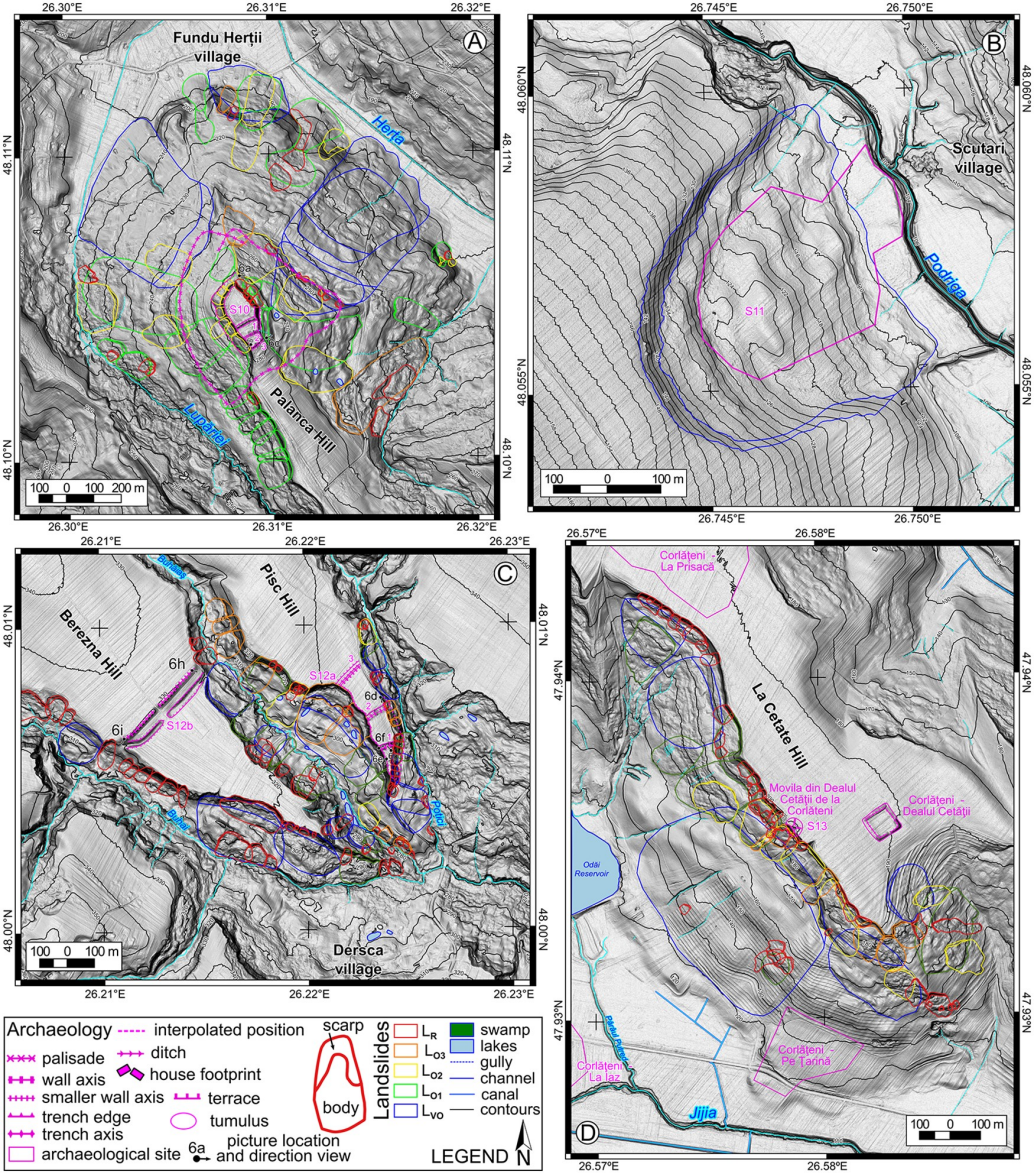
Landslide inventories and archaeological sites. (A) for site 10, (B) for site 11, (C) for site 12, and (D) for site 13. https://doi.org/10.6084/m9.figshare.11340419.

**Fig 3:**
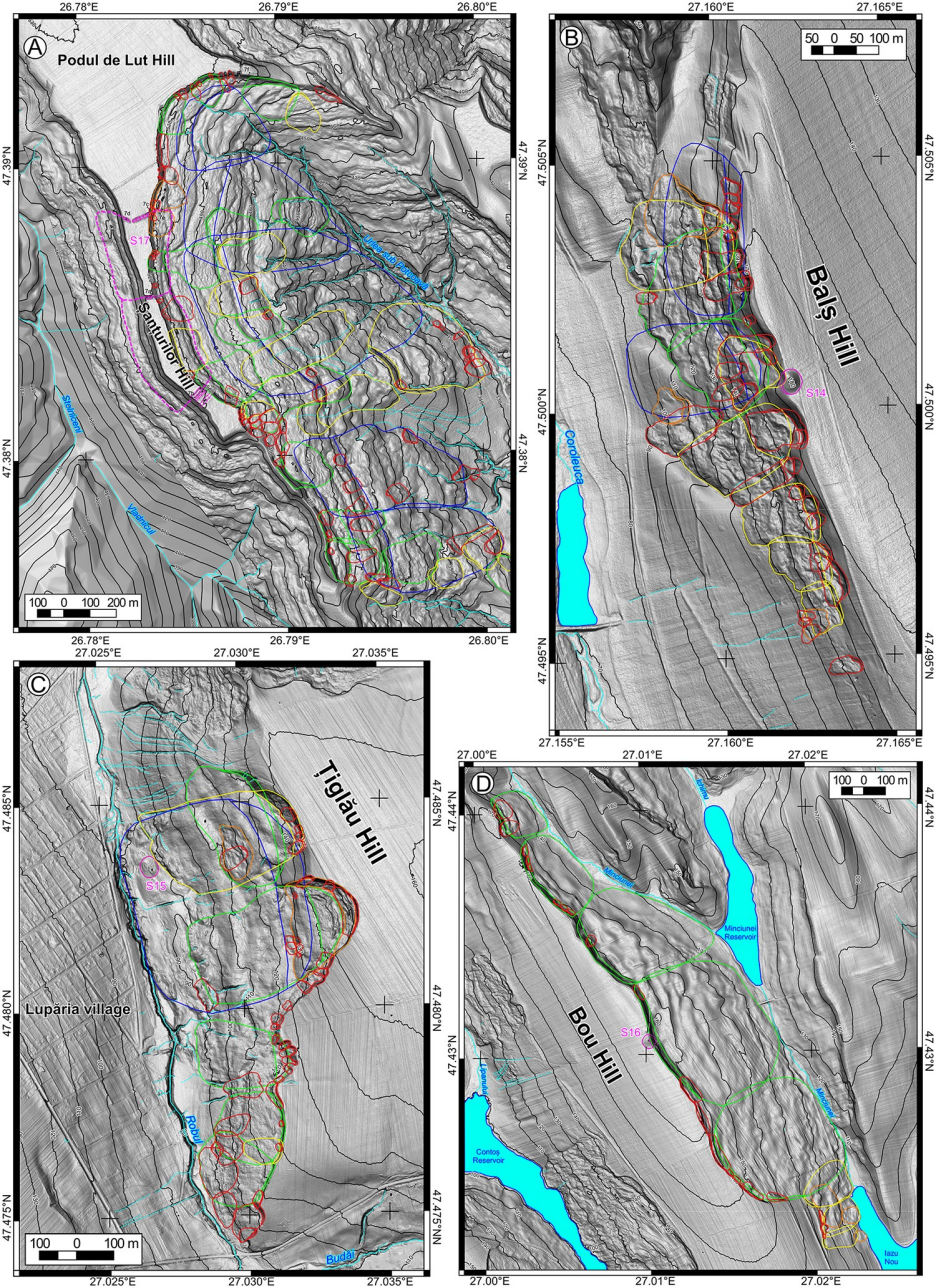
Landslide inventories and archaeological sites. (A) for site 14, (B) for site 15, (C) for site 16, and (D) for site 17. https://doi.org/10.6084/m9.figshare.11340425.v1.

**Fig 4:**
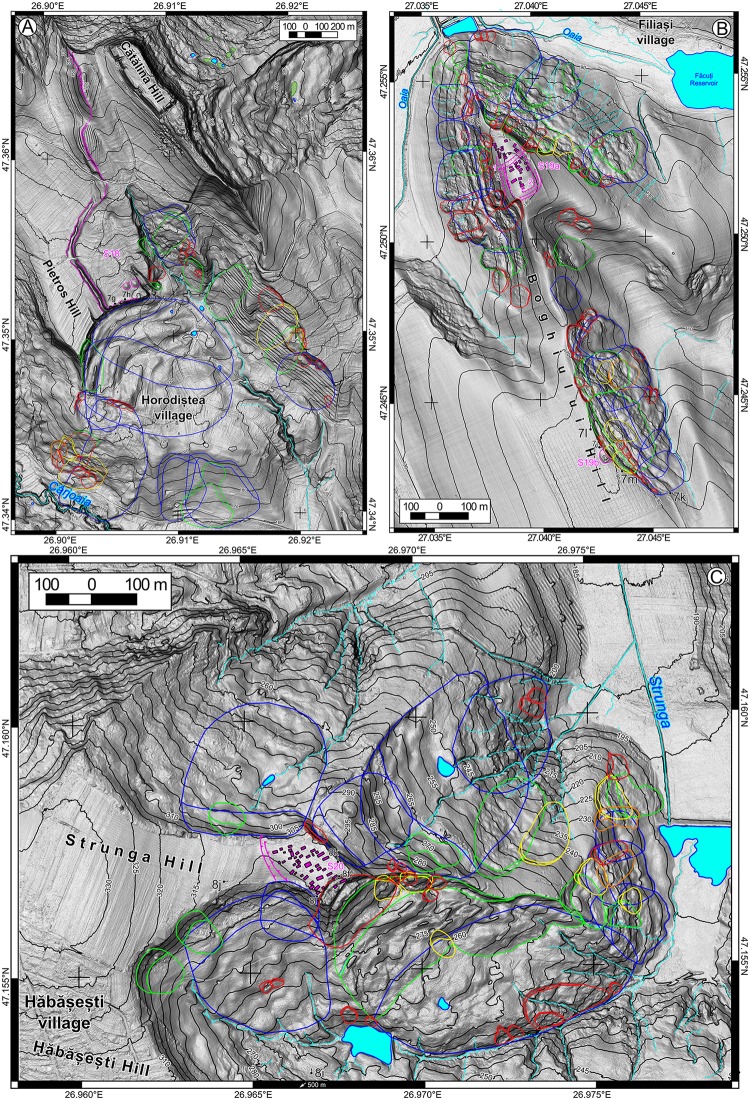
Landslide inventories and archaeological sites. (A) for site 18, (B) for site 19, (C) for site 20, and (D) for site 21. https://doi.org/10.6084/m9.figshare.11340428.v1.

**Fig 5:**
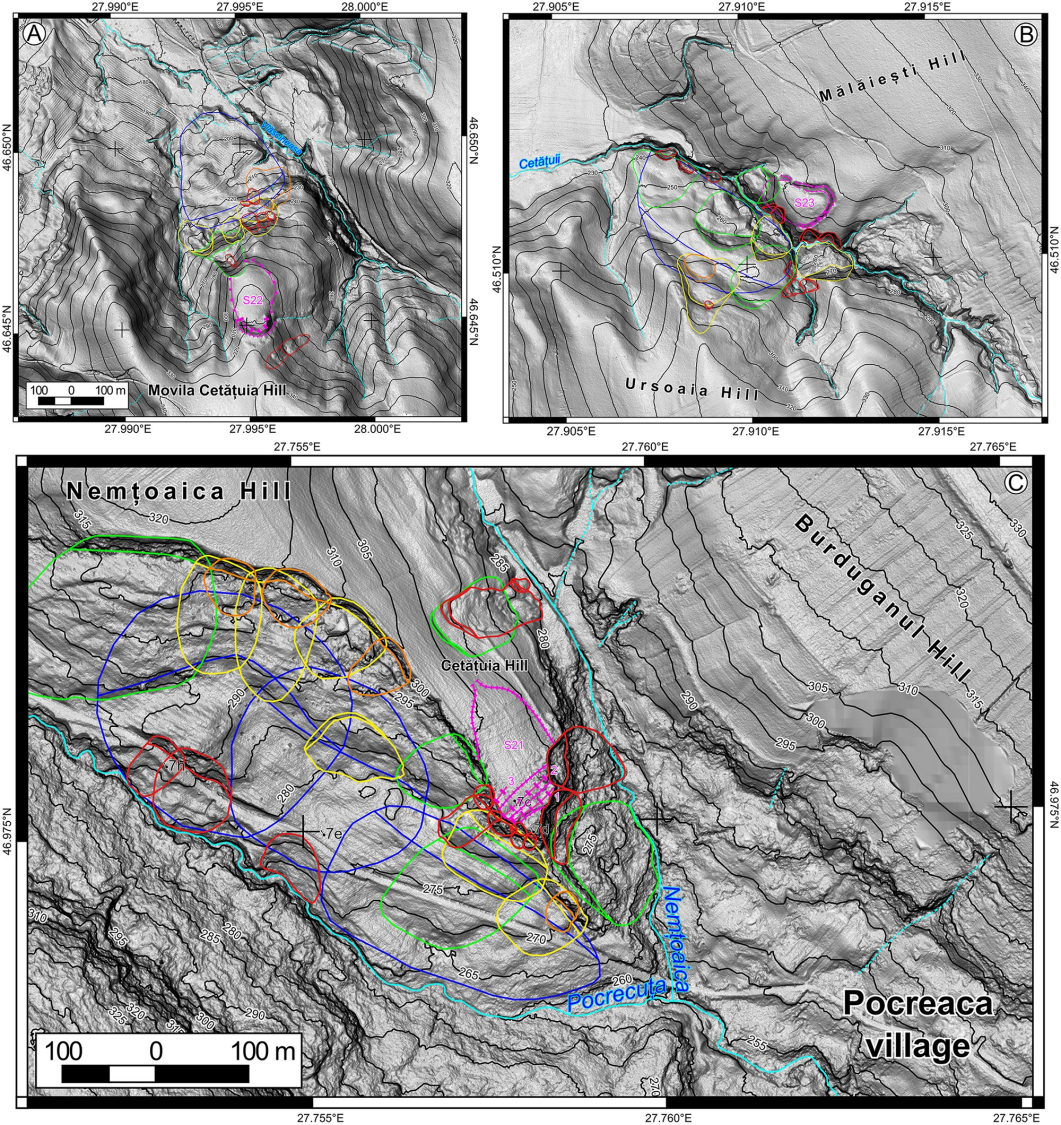
Landslide inventories and archaeological sites. (A) for site 22, (B) for site 23. https://doi.org/10.6084/m9.figshare.11340431.v1.

The landslide delineation methodology, which was applied in this approach is the same described in [39]. In the current work, we will detail the criteria of the landslide delineation. The main source based on which landslides were identified and delineated was represented by LiDAR data. The LiDAR data density is 2–6 points per m^2^, from which a 0.5 m resolution DEM was obtained using TIN interpolation method in SAGA GIS [84]. LiDAR represents one of the main sources of landslide delineation for various types of landslides [32,85–88] nowadays. Besides the high-resolution DEM, aerial imagery from different time periods (1954–2008), Google Earth imagery (2003–2017), topographic maps and DEM derivatives (topographic sections, contours, slope, shading, curvatures) were used to assess the local condition of the surface, in order to better understand the features recognizable on the DEM. The shading (background in Figs 2–5) was computed with various light source positions in SAGA GIS (Conrad et al., 2015). 3D anaglyph views generated in SAGA GIS were also useful for a better understanding of the landslide topography.

Recent landslides (LR) usually preserve all the landslide components [89]: the main scarp, the flanks, the toe, rugged topography, and are easily recognizable and delineable [90–94]. Besides the LiDAR appearance, aerial imagery provides useful information about the condition of the main scarp or minor scarps of the landslide body, which usually are not covered by or have little vegetation cover. The presence of cracks or fissures in the landslide body, scarp, and/or toe area suggests that the landslide is either still active or is very recent [95–98]. Old landslides (LO) still present the majority of the recognizable elements, although these are smoothed. In order to recognize the old landslides and to correctly classify them to a specific generation (LO1, LO2, LO3), subtle elements are needed to be identified. One of the easiest ways to recognize the topology of these generations is the case of a shallow slide affecting the landslide body of a rotation slide [42,60]. Often, because the new generations of landslides destroy the integrity of older generations of landslides, the initial extension of the landslide is interpolated using the curvature trend of the scarp or the bumps in the overlaying landslide body. Very old and relict landslides (LVO) are areas that present a concave surface mapped as scarp which is connected with a convex one which can be mapped as a toe and by a rough mass that also present on flanks a change in steepness (for mapping this areas the contours, the slope and curvature maps are useful–[87]. For the relict landslides, the old scarp location was interpolated using the morphological pieces of evidence obtained by topographic cross-sections. The presence of gullies on the landslide body or along the flanks represents another hint that the landslide is relict [91]. Although land use can induce variations of roughness (we have used aerial images to assess the roughness due to land use variations–[98], a relict landslide body still presents a roughness characterized by a wide spacing, which differentiates it from the smooth non-slided adjacent hillslope. In areas where bedding traces are present, the shallow relict landslides are difficult to delineate. Nonetheless, we avoided the delineation of the compound or complex landslides, as polygons which include all the rough areas where landslides appear from ridge to hillslope base and extended laterally along the hillslope, as for previous landslide inventories of the Moldavian Plateau [42,44]. Instead of assigning a level of uncertainty on landslide delineation, we preferred to extract only landslides that present the elements shown above, so having a high level of certainty about their presence.

**Fig 6:**
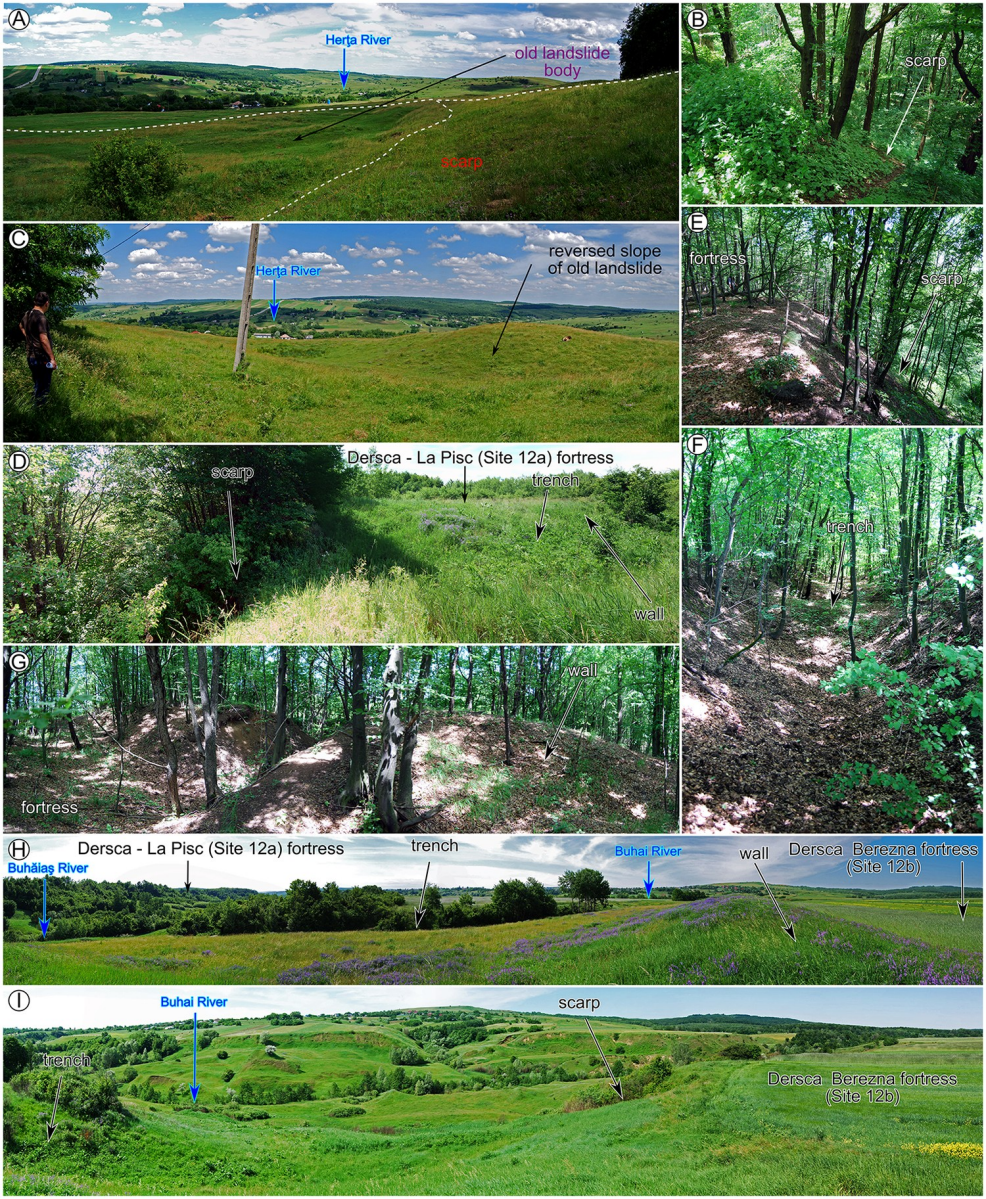
Pictures showing field checked relations between archaeological sites and landslides. (A-C) for site 10, (D-G) for site 12a; (H and I) for site 12b. https://doi.org/10.6084/m9.figshare.11340458.v1.

**Fig 7:**
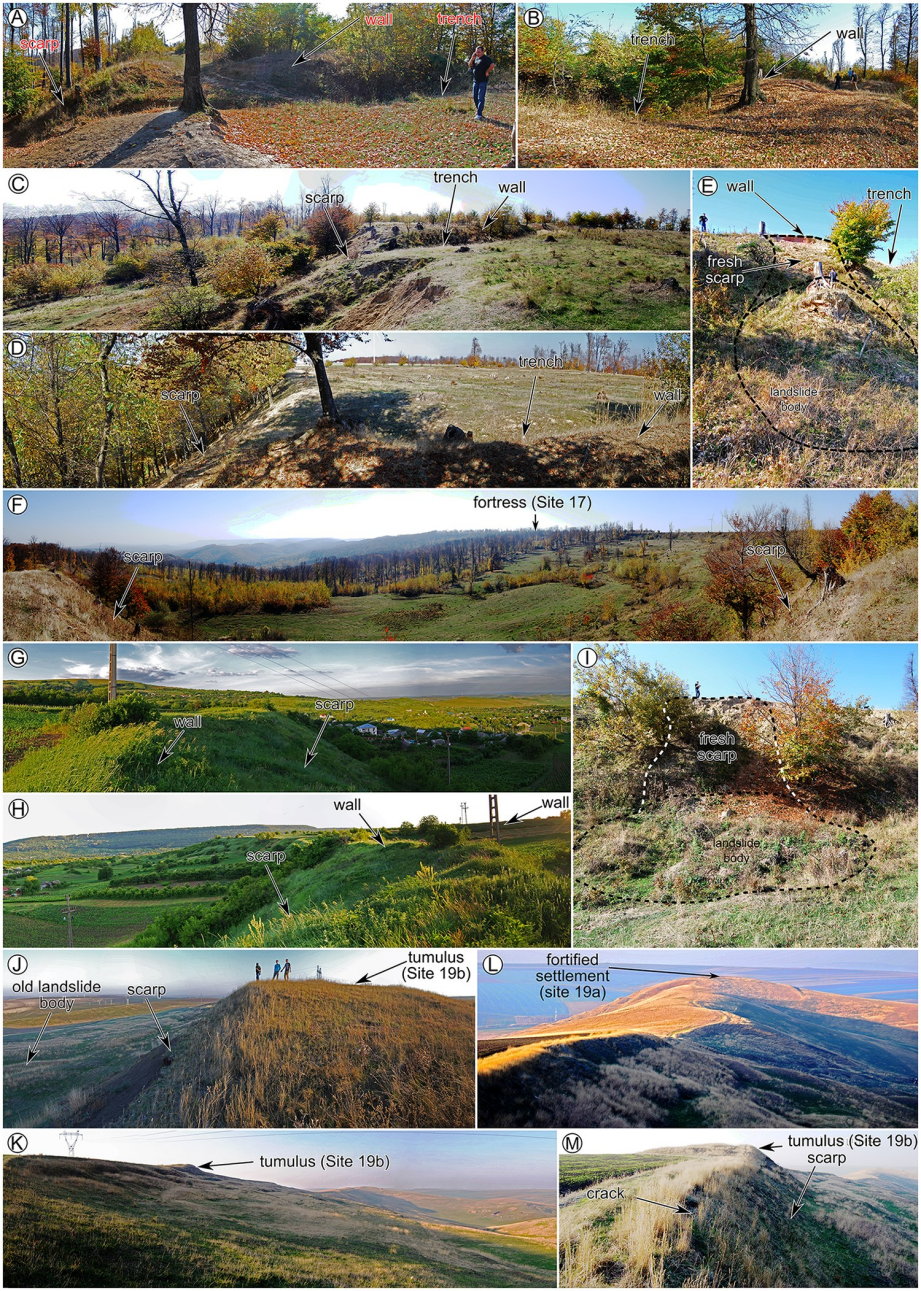
Pictures showing field checked relations between archaeological sites and landslides. (A-F and I) for site 17, (G and H) for site 18, (J, K, M) for site 19b, (I) for site 19a. https://doi.org/10.6084/m9.figshare.11340485.v1.

**Fig 8:**
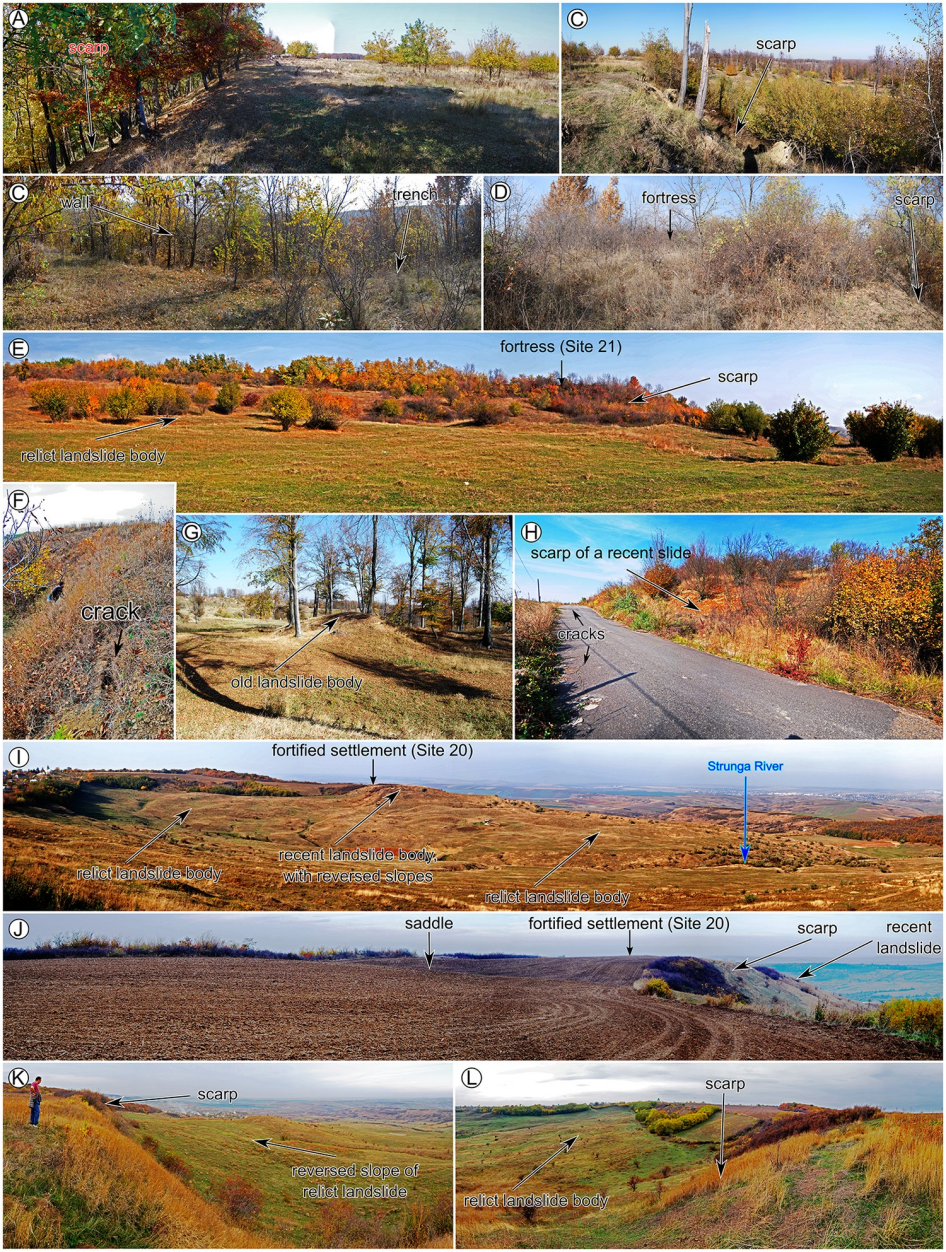
Pictures showing field checked relations between archaeological sites and landslides. (A, B and G) for site 17, (C-E and H) for site 21, (F, I, J, K, L) for site 20. https://doi.org/10.6084/m9.figshare.11340449.v1.

After the inventories were carried out, the field check was performed to validate the inventories (photography from the filed check are shown in Figs 6–8), and adjustments were done where the field evidence required it. For the fieldwork there were no specific permissions required, because all the areas are in public domain and the field check was done through visual reconnaissance. The landslides were mapped as polygons, and the attributes regarding the relative age (relict, three generations of old and recent landslides) were assigned in QGIS software. After the attribute assignment, the polygons were split by a line to separate the scarp from the landslide body. Landslide type was assessed according to [89] classification as earth falls, earth flows, rotational slides, and translational slides.

### Landslide area distributions

**Fig 9:**
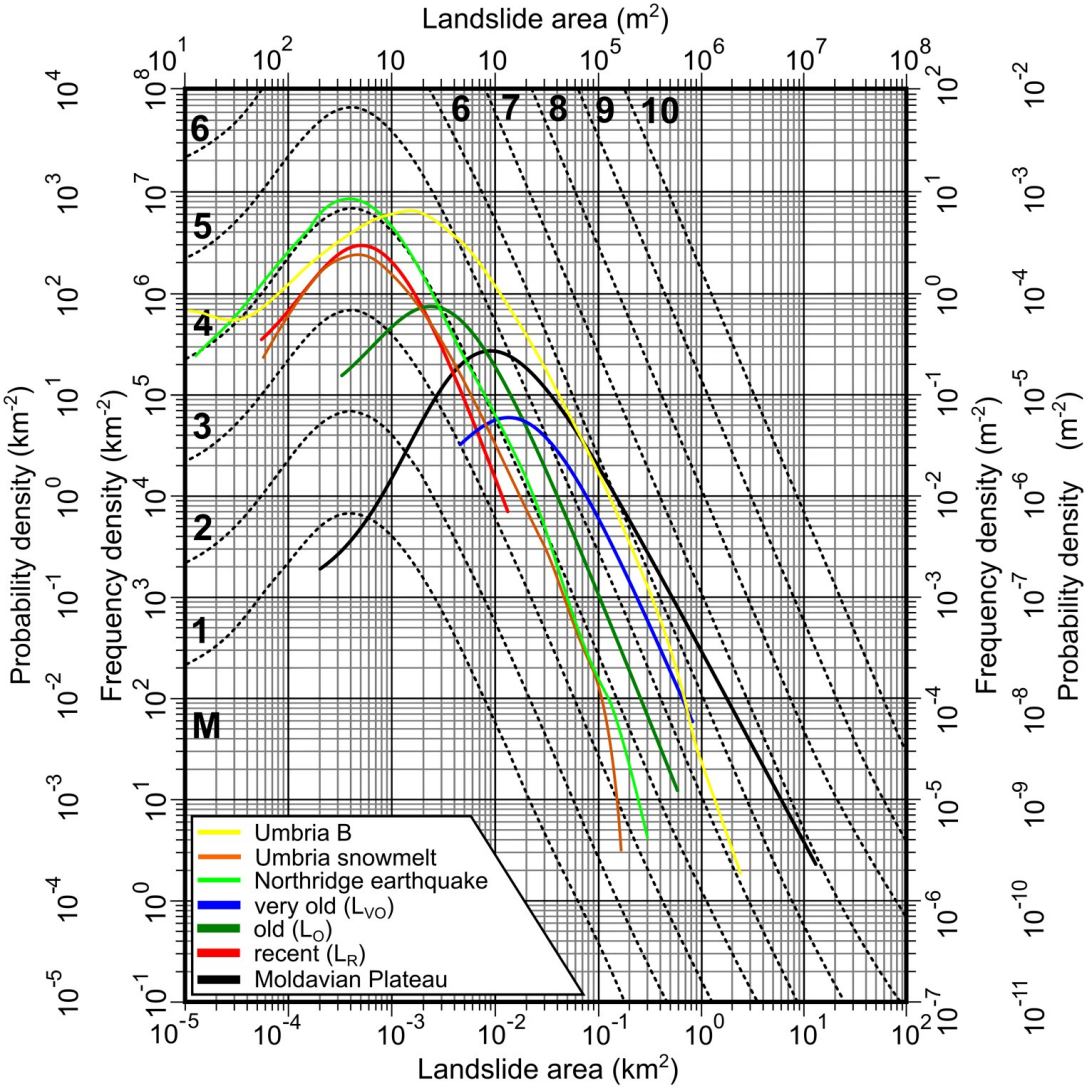
Landslide size distributions grouped by relative age classes for the landslide inventories of [39] and from the present work. Umbria and Northridge data are taken from [100] and the Moldavian Plateau data is taken from [42]. https://doi.org/10.6084/m9.figshare.11340488.v1ERT.

To test the validity of the produced inventories and to test if the different relative age landslides follow magnitude curves as it is stated in the literature [36,99–102], we computed the landslide frequency density curve (Fig 9) using LANDSTAT R stat [103] script developed by [104].

**Fig 10:**
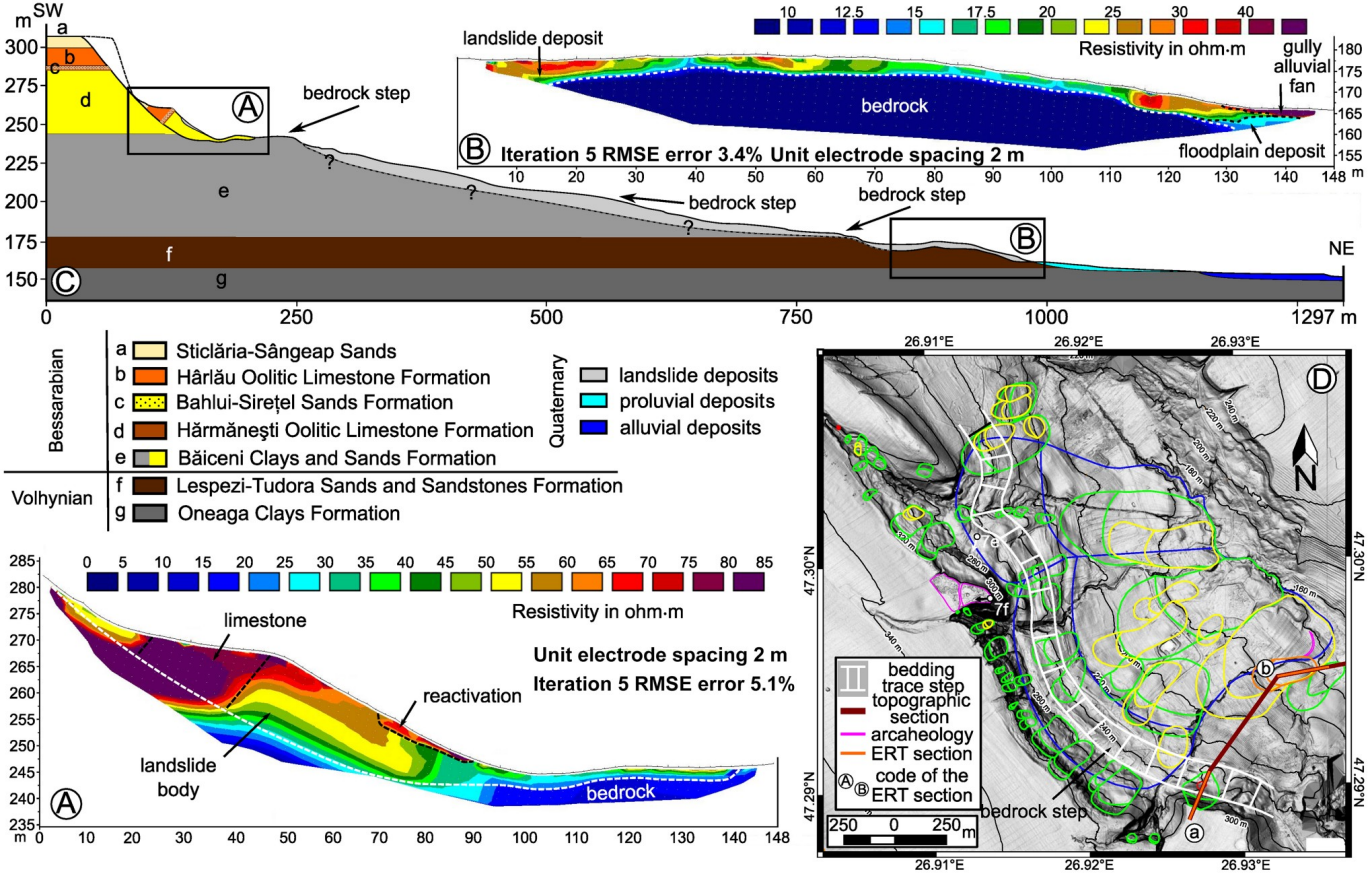
Băiceni Hillslope landslide geomorphology. (A) ERT section of the scarp reactivation, (B) ERT section of the toe and Băiceni River floodplain [55], (C) geomorphologic profile (the geology is after [53], (D) Băiceni Hillslope landslides and archaeology [39] with the location of the topographic section and the ERT sections. https://doi.org/10.6084/m9.figshare.11340494.v1.

The Electrical Resistivity Tomography (ERT) method is based on the capacity of the subsurface to resist on an electrical current [105,106], highlighting horizontal and vertical dimensions of ground properties, especially in the case of significant contrast in resistivity of the structures [107]. ERT is known as a method with which landslide slip surfaces can be identified, so consequently, the geometry of the landslides can be understood [108–114]. The resistivity can indicate the presence of clay minerals, a high degree of fracturing and saturation with water, which usually gives low resistivity (under 100 ohm-m), compared to hard rock, unfractured, or unsaturated water conditions, which usually have high resistivity [115,116]. When claystones are weathered and contain water, they can have smaller resistivity values [117,118]. The main advantage for landslide analysis using ERT is that the slided material has very often a low lateral and vertical variation in resistivity [112], or the disturbance creates a non-homogeneity, which contrasts with the homogeneity of the bedrock [105,107]. Although in general ERT results are confirmed by other geophysical methods [119], there are situations where ERT fails to deliver an answer for the sliding surface because of the similarity of the clay content of the slided mass and the bedrock [120]. We surveyed the Băiceni complex landslide [39,55] which we considered a representative site for all the generations of landslides to understand it’s geometry in the scarp and the toe area with two ERT cross-sections (Fig 10). A linear profile was designed along a representative path at the upper and the lower part of the hillslope. The measurements were carried out using a GeoTom MK1 resistivimeter equipped with 50 stainless steel electrodes, equally spaced at 2 m. For each cross-section, the apparent resistivity data were acquired using the Wenner array and a roll-along technique. The relative three-dimensional location of each electrode along the ERT profiles was accurately recorded in the field using a Leica TC407 total station, and topography was included in the inversion routine. The Wenner array was preferred due to its good signal-noise ratio and moderate penetration depth [121], up to 16 m in the case of this study. Moreover, it offers moderate sensitivity for the detection of both horizontal and vertical subsurface changes [122]. To transform the apparent resistivity pseudosections into 2D models of calculated electrical resistivity, we used Res2Dinv software [123] and a robust constraint inversion method, less sensitive to very noisy data points and more suitable for the identification of the sliding surfaces.

## Results and discussion

The landslide inventories and archaeological topography are depicted in Figs 2–5. In the following sections, we will discuss every site and emphasize the relation between the archaeological topography and the mapped landslides (detailed descriptions of the archaeology and geomorphology of every site can be read in S1 Annex). In Figs 6–8 there are field photos that pinpoint the discussed relations.

### Hilltop sites

The majority of the described sites fall in the category of hilltop sites (Sites 10, 17, 19a, 20, 22). These areas were favorable for settlement construction because of the flat hilltops (structural plateaus from a geomorphological point of view) bordered by escarpments which provided the advantage of inaccessibility and the ability to have a wide view on the surrounding areas. Often gully or river incision and/or the landslides triggered by these incisions generated promontories that were easy to protect trough the construction of a wall and trench system toward the narrow gentle slopes of the plateaus (Site 21) or hillslopes (Sites 12, 24).

Fundu Herţii medieval hillfort (Site 10) and its previous Chalcolithic settlement used a secondary hilltop whose edges were bordered by relict landslides (Fig 2A). Recent landslides (post-medieval) have affected the North-Eastern part of the fortress, the landslide scarp retreat being estimated at 30–40 m in this location.

La Pisc medieval fortress (Site 12) used the flat plateau created by the incision of local rivers (Fig 2C). The river incision also triggered the valley side retreat through various generations of old landslides, maintaining the vertical shape of the hillslope near the plateau edge. Recent landslides (post-medieval) have affected the North-Eastern part of the fortress, the landslide scarp retreat being estimated here at 30–50 m. The Berezna site has a similar landslide induced morphology with the difference that the old generation of landslides affected mainly the area around the confluence of the Buhai River with the Buhăiaş River, and not the edges of the thraco-getic enclosure, which is not affected by recent landslides (Fig 2C).

Corlăţeni site (Site 13) is an interesting case of post-roman period landslide events. At the base of the hill, there is a settlement that was populated during Chalcolithic and post-Roman periods (Fig 2D). The tumulus mound, built by Bronze Age populations, was affected by recent landslides, probably after the 2350–2250 yr. BP. The medieval fortified settlement built on the plateau is located at a safe distance (240 m) from the hilltop edges, which were probably affected by landslides at that time.

Todirești site (Site 17) is an example of thraco-getic hillfort affected by old and recent landslides that used a flat hilltop bordered by escarpments generated by very old landslides (Fig 3A). Through the analysis of the topographic cross-sections on LiDAR DEM the retreat of the plateau is estimated to be between 60 and 150 m.

Horodiștea is a site (Site 18) where relict landslides created escarpments which allowed natural protection along the Southern edge of the Cătălina fortress (Fig 4A). The western flank of the hillfort was protected by the Horodiștea wall and trench system, built laterally of the Cătălina Hill.

The Filiași–Dealul Boghiului Chalcolithic settlement (Site 19) is located on a narrow and relatively flat secondary ridge (Fig 4B). The upper part of the eastern and western hillslopes of this secondary hill are relict (very old) landslide scarps, which created natural protection for the settlement. Old and recent landslides have destroyed a part of the settlement, especially on its North-Eastern part. We estimate that the linear scarp retreat here was between 30 and 60 m, while in the southeastern part, the retreat was estimated to be under 30 m. The Northern edge of the ridge has army trenches that were identified on aerial imagery (taken in 1959) and which at that time extended from 5 to 10 m to north compared to the present-day situation. A recent landslide that happened somewhere between 1945 and 1959 affected this area (Fig 4B).

Hăbășești site Chalcolithic settlement (Site 20) is located on a rectangular flat ridge bordered by relict landslide scarps (Fig 4C). The eastern scarp reactivated in 1930 [81] and shows the mechanism of a rotational slump (Fig 4C).

Pocreaca site (Site 21) is similar to the Chalcolithic sites studied in 2016 [39], here the Chalcolithic settlement and the thraco-getic hillfort used a hilltop bordered by relict landslide scarps (Fig 5C). The scarps reactivated on the southern flank in old and recent times, generating scarp retreat of around 20 to 60 m.

Creţești (Site 22) and Corni-Albeşti–Vladnic (Site 23) sites are similar to the thraco-getic sites studied in 2016 [39], the locations being favored by relict landslides and being affected by old and recent reactivations. For Creţești the northwestern scarp old slump (Fig 5A) generated a scarp retreat of 30 m. For Vladnic the northwestern scarp old slump (Fig 5B) generated a scarp retreat of 50 m. In both locations, the steep areas of the hillslope where wall and trench systems could not be built were protected by using a terrace (Fig 5A and 5B) on which probably wood palisades were built, a situation similar with Moşna fortress [39].

Scutari site is an open Cucuteni period settlement located on a relict landslide body (Fig 2B), similar to the Băiceni site [39].

### Tumuli (burial mounds)

Besides fortresses, hilltops also hosted the location of burial grounds, which consist of tumuli mounds for certain cultures (Yamnaya, scitians, sarmatians). The majority of the tumuli from Jijia Hills are located on ridges (Corlăţeni–[77]; Movila Carului from Şuletea–[124]), but there are also cases of tumuli located on hillslopes (La Stadole–[76]), lower fluvial terraces (Valea Lupului–[77]) and floodplains (Glăvăneştii Vechi–[76]). The majority of these tumuli are Yamnaya graves [76,77,124,125], only a few being of sarmatic origin [76]. In the northern part of the studied area, at Glăvăneştii Vechi [77], Truşeşti [126] or Roma [125,127] old (probably Yamnaya) tumuli were used for burials in later periods, including Hallstatt, Sarmatic (1850–1650 yr. BP) or Turanic periods (950–650 yr. BP).

For the Central and Southern part of the Moldavian Plateau, the archaeological literature has shown that two main periods are characterized by tumuli construction. The first is the Bronze Age period, during which the Yamnaya shepherds built burial mounds in the steppic areas of Eurasia and Eastern Europe. The Yamnaya burial mounds, also called Pit-Graves appear in Eastern and Southern Romania. While in Eastern Romania, there is no 14C dating, data from Southern Romania [128] or other parts of Eurasia [129] framed this culture in the period of 5500 to 4400 yr. BP. In the Moldavian Plateau, Yamnaya burial mounds are very dense, especially in the Jijia Hills. The second period is 1850–1650 yr. BP, when especially in the Central and Southern part of the Moldavian Plateau the Sarmatic populations also built burial mounds. In the Jijia Hills, it seems that the Sarmatic populations mainly reused old Yamnaya tumuli, which were very dense and occupied the most favorable ridges and plateaus [74].

The majority of the Yamnaya Bronze Age tumuli mounds are located on hilltops, either on the highest point or close to the plateau edge, to be visible from the surrounding areas. Although smoothed in the present-day topography, these mounds are still visible in the field from the surrounding areas. The fact that these mounds were located on plateau edges, which were bordered by relict landslide scarps, shows that during the Bronze Age period, those scarps were inactive. The fact that these mounds were also used by 1850–1650 yr. BP Sarmatic populations for burials indicate that even in that period these locations were considered safe concerning their proximity to landslides. In our view, the old and recent landslides which affect these mounds should be considered medieval and post-medieval.

The most informative site from this point of view is Corlăţeni tumulus mound (Fig 2D). Since the landsliding processes affected the mound, we argue that the age of the landslide is recent, long after the Yamnaya mound was constructed. At the same time, it is clear from the localization of the tumulus that the Corlăţeni mound was constructed at a certain distance from the ancient edge of the ridge, and that through retrogressive mechanisms, the post-Yamnaya scarp advanced gradually. The analysis of the topography of the scarp from this area (Fig 2D) shows a maximum retrogression of the scarp to be around 200 m.

The other three sites with plateau edge tumuli mounds affected by landslides (Sites 14, 16 and 19b –Fig 3B and 3D, 4B) have relict landslides on the hillslopes, but at the moment of mound construction, their scarps were at a safe distance. Only later, the landslide scarp reactivated and generated a retreat and affected the mounds. The Balș mound (Site 14) is affected by old landslides, which generated a scarp retreat of around 40–50 m (Fig 3B). The Bou mound (Site 16) is affected by old landslides that generated a scarp retreat of around 180 m (Fig 3D). The Boghiului mound (Site 19b) is affected by old landslides that generated a scarp retreat of around 60 m (Fig 4B).

The presence of Bronze Age tumuli mounds on relict landslides bodies, like in Corlăţeni, La Stadole and Lupăria (Fig 3C) areas [74] emphasize the Lower Holocene Age of the relict landslides.

### Magnitude distribution of landslide inventories

Adding the new mapped 702 landslides to the 509 landslides mapped by [39], we obtained a landslide inventory containing 1211 landslides. From these landslides, 118 are very old (relict), 627 old and 444 recent. The frequency-area distribution of landslides for every relative age category was computed (Fig 9) for the created inventories, and it is consistent with the results from other regions over the Globe. All the inventories are not complete, the recent landslides inventory having a distribution shape similar to event-based inventories. The inventories are geomorphological, showing a lack of small landslides, which either disappeared from the morphology or are not present because the area where the landslides were mapped does not cover a representative area [99,100,102]. Nonetheless, the tail of the curves follows the trend of theoretic and verified landslide area probability density distributions [36,99–102]. The relict landslides fit the magnitude 6 distribution (blue line), the old landslides magnitude 4 to 5 (dark green line) and recent landslides magnitude 4 (red line) showing a decrease in magnitude over the time. This evidence of magnitude decrease strengthens the conclusions of [39] regarding the validity of the landslide delineation criteria and of the obtained landslide inventories in representing temporal different landslide events.

### Landslide geometry based on ERT

The two ERT surveys were used to test the validity of the geomorphological analysis on LIDAR data (Fig 10). We performed these geophysical investigations for Băiceni site which was studied by [39] and where relict landslides have possibly Upper Pleistocene age [41].

For Băiceni site we investigated the toe and the scarp of the relict landslides. The toe section shows (Fig 10A) the presence of a 5 m thick landslide deposit with high resistivity over the siltstone-mudstone bedrock (Oneaga Clays Formation), which presents low resistivity and is homogenous. The high homogeneity could be explained better by the high clay content and a lack of fractures rather than by water saturation conditions. The landslide deposit sits on the floodplain of Recea (Băiceni) River and is not so homogenous in resistivity compared to the bedrock and the floodplain deposits. The scarp section targeted an area where there is a visible old landslide not delineated in [39] because is lateral to the main archaeological site. This landslide was also affected by a recent reactivation. Both landslides deposits (the old landslide and its recent reactivation–Fig 10C) can be recognized on the ERT section (Fig 10B), and the internal geometry of the old landslide can be depicted: (i) the oolitic limestone layer present high resistivity; (ii) the sands from above or below this hard layer have medium resistivity; (iii) the boundary between the landslide deposits with medium resistivity and the Băiceni Clays and Sands Formation with low resistivity can be traced along the 50 ohm-m value; (iv) the basal part of the landslide had mobilized also a part of the clays, and here the sands spread on the bedrock (Fig 10C and 10D); (v) the recent landslide deposits have a high resistivity compared to the old landslide body deposits. The old landslide is a rotational rock slide (slump) of type 6 according to [2] classification, while the recent landslide is a planar earth translational slide of type 12 according to the same classification. The fact that the Bahlui-Sireţel Sands are cohesive allowed the rock mass to slide and remain relatively undisturbed, without collapsing too much (except for the toe, where landslide deposits have spread over the underlying bedrock step—Fig 10C and 10D). The presence of the continuous lateral steps in the hillslope morphology influenced by the bedding traces intersection with the surface, between the scarp and the toe for the complex Băiceni Hillslope landslide is a sign that the relict and old landslide deposits are thin (because we do not have ERT data in that area we represented them with question marks in the geomorphological profile from Fig 10) and sometimes smoothed by erosion or deposition events [39,55]. The presented evidence shows strong support for the hypothesis that hillslopes like Băiceni site are complex landslides that evolved during the Holocene by retrogressive reactivations of the scarp and by continuous sliding of the upslope material toward the initial toe of the previous landslides. This type of evolution created stratified deposits, which are characteristic for many hillslopes from the Moldavian Plateau [42,55].

### The pattern of landslide spatial and temporal evolution

In the central part of Romania (the Transylvania Depression) there is a long record of dated landslide events using the age of damned lakes along river valleys: Măgheruş Valley–landslide events at ~17730±165 cal BP and ~ 15 300 kcal BP [130] or of lakes placed on top of large landslide bodies: using the basal organic layer of Tăul fără Fund peat bog the Pădureni landslide was dated to be pre 1820±30 y BP [131].

In the Eastern Carpathians of Romania there are also several dated landslides related to valley damned lakes: Bolătău landslide (23 ha, 9 mil. m3 [132]) who’s age can be constraint to 6.8–7 ka BP period [133], Iezer landslide which is 950 y old [134], and the Red Lake landslide (22.5 ha, started in 1837–1838 with reactivations in 1953–1959, 1978–1979,—[135]). Similar ages were obtained in the Czech Flysch Carpathians by [66] for landslide dams.

In the Western Carpathians, [136] and [137] determined the climatic variability of landslide activity, starting with Older Dryas and continuing in Allerod, Boreal, Atlantic, Subboreal, Subatlantic and in the last 100 years. A similar pattern was argued by [39] for the Eastern Carpathians’ lowland, with continuous Holocene activity and possible Lateglacial events [41]. Usually, the pre-Holocene landslides are identified only from deposits, their morphologic signature being disappeared (especially in the mountainous area), but there are notable exceptions from the hilly area of Central and Eastern Europe [9,138,139].

**Fig 11:**
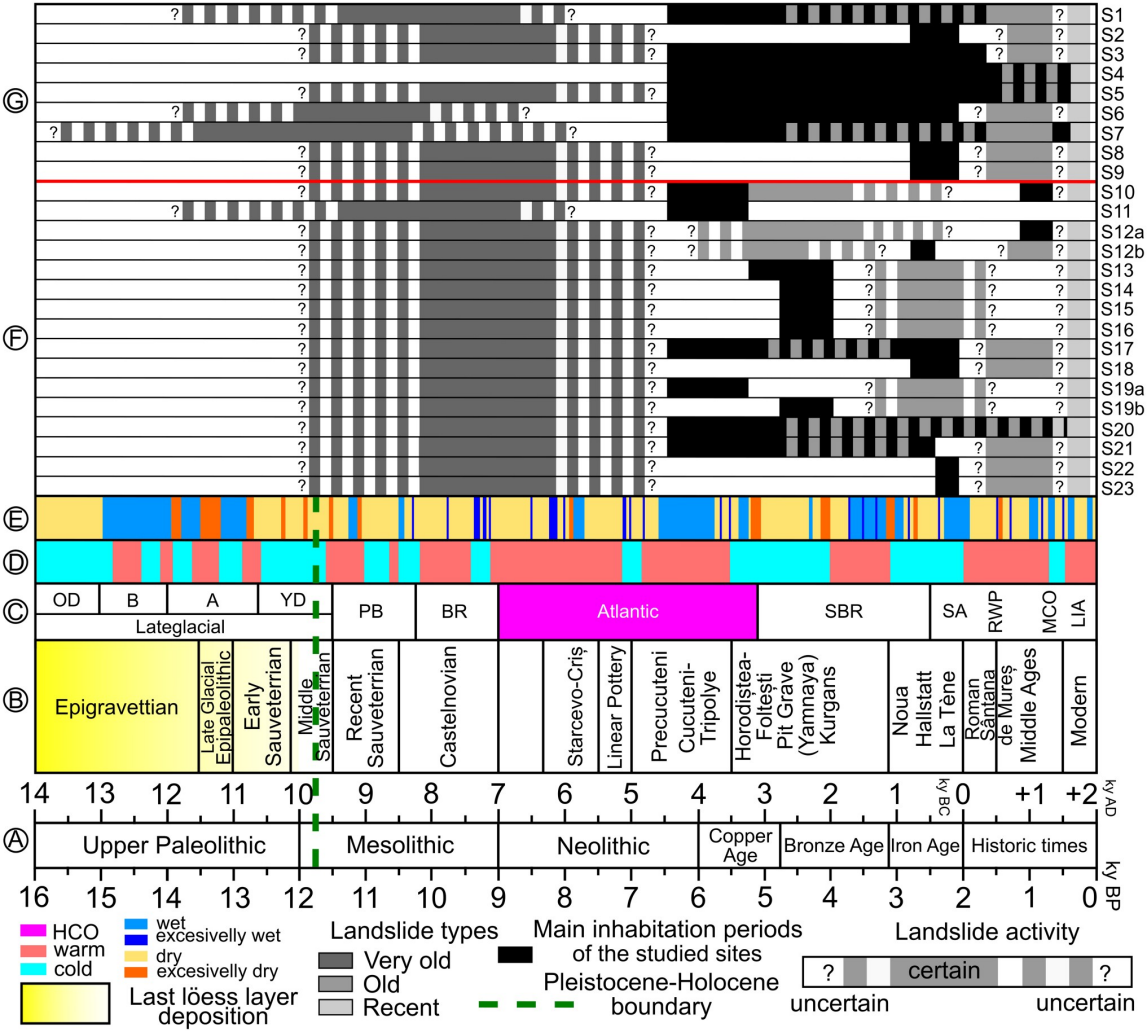
Synthetic representation of the Lateglacial to Holocene chronology [140,141], archaeological and climatic environment in the context of landslide activity. (A) human prehistory and history chronology(synthesized by [39] with slight modifications considering the advancements from [41], (B) human cultures in prehistory and history of Eastern Romania (synthesized by [39], (C) Blytt-Sernander classification system ([142] matched to Romania dates using information from [140]) (B–Bølling, OD–Older Dryas, A–Allerød, YD–Younger Dryas, PB–Preboreal, BR–Boreal, SBR—Subboreal, HCO–Holocene Climatic Optimum, SA–Subatlantic, RWP–Roman Warm Period, MCO–Medieval Climatic Optimum, LIA–Little Ice Age), (D) paleoclimatic reconstructions for Romania of wet vs. dry periods [67,68,140,141,143–145], (E) paleoclimatic reconstructions for Romania of warm vs. cold periods [140,141,143], (F) landslide activity for the studied sites (numbered according to Fig 1 and Table 1); (G) landslide activity for the sites from [39] (numbered according to Fig 1); Pleistocene to Holocene boundary is taken from [146]. https://doi.org/10.6084/m9.figshare.11340497.v1.

Although no absolute dating of landslides was done yet in Moldavian Plateau, mainly because of the lack of proxies (landslide body lakes are present, but the climate didn’t favor the bog formation, and seasonally these lakes might disappear), archaeological and geomorphological evidence allow us to draw a general timing of landslide activity and to assign relative age to the mapped landslides. The very old/relict landslides have Lower Holocene (Atlantic period) or even Upper Pleistocene (Lateglacial) age (Fig 11) [41], as in other hilly areas from Europe [30]. This period was shown to be cold and dry in Lateglacial and warm and wet in the Lower Holocene (in Bukovinka cave, which is the closest proxy, for the Lateglacial is known for frequent soil disturbances and Lower Holocene by the lack of deposits showing an increased erosion). Such conditions probably favored massive landslides triggered by the LGM incision of the river network, by the lack of forest vegetation and by wetter periods. Considering the last wet period from the 70’ and the 80’ an increase of the mean annual rainfall amount with 100–200 mm is enough to increase the frequency of the landslide events [42,61]. The intense population from the Chalcolithic period, from the Holocene Climatic Optimum (Atlantic), when the climate was warmer, but dryer could show a decrease of the landslide activity. During this period, the inaccessible sites created by relict landslides were heavily used as defensive sites by the ancient populations.

The old landslides appeared on an interval that ranges from the Atlantic period to Subboreal and Subatlantic. The end of the Atlantic period is known to present climatic changes, which also influenced the Cucutenian culture. The other cyclic disturbances which appeared after 6 ka yr. BP can be the trigger of the different generations of old landslides. These also affect the thraco-getic hillforts, which show that their last generation happened even after 2.5 ka yr. BP, a period in which the present-day vegetation started to develop. In this context, the recent landslides are post 1 ka BP and appeared under the influence of the increase in forest clearance and the cold and wet periods of the Medieval Period [147].

The geophysical investigations of the Băiceni complex landslide coupled with the knowledge gathered during the inventory creation and field validation makes us argue the following conceptual landslide evolution pattern: (i) during the Lateglacial and the Lower Holocene the hillslopes were destabilized by the river incision and by incipient big landslides, considered now as relict; (ii) after the Holocene Climatic Optimum, different wet periods triggered old generations of landslides which reactivated the relict landslide bodies and scarps, generating a predominant retrogressive mechanism of landsliding and maintained the scarps steep and generated the multilayered stratigraphy of slope deposits; (iii) after the Medieval period, the cold and wet periods generated the recent landslides;(iv) both new generations of old landslides and the recent landslides generated the destruction of tumuli mounds and thraco-getic fortifications which were close to the scarp edge; (v) the landslide event magnitude decreased toward the present day, both because of the decrease of climate variability and because the scarps and the slope deposits are much more susceptible to reactivations than to huge events. This pattern of temporal evolution generated complex landslides, which sometimes cover the hillslopes of the Moldavian Plateau on several kilometers and represent areas with a continuous landslide occurrence over the Holocene and where recent reactivations predominantly occur [42,148–150].

As a general observation for our landslides inventory, we can affirm that deep-seated and rotational slides are not so frequent as the translational slides. The presence of steep hillslopes on the monoclinic structure with an alternation of bedded mudstones, claystones, sands, siltstones, sandstones and limestones, where the slopes are mainly anaclinal (with steepened, normal or subdued escarpments, sensu [151]) or ortoclinal, generates the vast extension of translational slides. These slides overlay each other and in time, generate complex landslides morphologies that can span several kilometers along the cuesta escarpments [42]. If the landslides are shallow and develop in a retrogressional manner the hillslopes topography will be dominated by continuous lateral steps under the influence of the bedding traces intersection with the surface, which generates a terraced like topography, especially in the scarp area of the complex landslides. This topography led [152] and others [153] to wrongly assume that these landslides are deep-seated or rotational slumps or block slides. The bedding traces can be followed sometimes on several kilometers, having morphologic signature even on non-slided hillslopes, and are interrupted only were rotational slides or earth flows develop or when are covered by landslide bodies.

This general pattern of the landslides from the Moldavian Plateau is very similar to other regions with monoclinal geological structures, like in Franconian and Svabian Alb, Germany [64,65,154] or Crimea [66].

### The relevance of landslide chronology building

The relevance of the presented approach is related mainly to the usage of the landslide inventory. The landslide inventory is the main tool through which geoscientists investigate beside the evolution of landforms dominated by mass-wasting processes [15–19], the distribution, types, pattern, recurrence and statistics of landslides, evaluate landslide susceptibility, hazard, vulnerability and risk [102]. If the landslides inventories have beside information about the magnitude, the temporal component, the hazard can be estimated. The landslide inventories that were obtained show clear association between relative age and magnitude distribution, so can be used for hazard estimation. The estimation of hazard and risk is important for establishing management strategies of the landslide risk [155], and has a socioeconomic significance. Since these landslide inventories cover the area around archaeological sites, the first application can be the hazard and risk estimation for these important sites. The relative age also can be used in geoheritage assessment [43].

## Conclusions

In the present work, we have shown a method of constructing relative landslide chronologies based on the relation between mapped landslides (landslide inventories) and archaeological site topography. The study case is a monoclinic hilly region (Moldavian Plateau), which showed an extensive chronology of landslides during the Holocene, and for which we presented a pattern of landslide evolution sustained by geophysical data and landslide inventories. The presence of fortified settlements from the Cucuteni culture, thraco-getic period (Iron Age), and the early medieval period together with the Bronze Age tumuli allowed us to establish topological relations with the landslide morphology on high-resolution DEMs. The landslide inventories were validated using landslide area density distribution, and their analysis shows a consistent landslide magnitude decrease from the Lower Holocene to the present day. Relict (very old) landslides have Lateglacial to Lower Holocene age, the old landslides have post HCO and pre-Medieval age, while the recent landslides have post Medieval age. ERT and geomorphological mapping provided information on landslide geometry, arguing for a retrogressive landslide mechanism regarding the evolution of the landslides from the Moldavian Plateau during the Holocene. This continuous evolution created complex landslides morphologies and layered slope deposits. A further analysis that might date absolutely with radiocarbon organic material from bellow, inside and from above the landslide, will improve the proposed chronology, but still, the presented methodology could be applied to densify the spatial coverage of landslide age information. We consider that the methodology could be applied for establishing relative chronologies of landslides in other regions if these elements are present: a high-resolution DEM for landslide inventory creation, investigated and dated archaeological sites, and a well understood landslide evolution pattern.

## Supporting information

S1 Annex Archaeological sites description and geomorphology. (DOCX)
