# Supplementary material for: Using archaeological and geomorphological evidence for the establishment of a relative chronology and evolution pattern for Holocene landslides
Source: PLoS One. 2019 Dec 31;14(12):e0227335. doi: 10.1371/journal.pone.0227335 (PMC6938354; doi:10.1371/journal.pone.0227335)
Supplement: S1 Annex — (DOCX) [file pone.0227335.s001.docx]

**S1 Annex 1 - Archaeological sites description and geomorphology**

**Site 10: Fundu Herţii – La Redută**

The Fundu Herţii – La Redută site (S10, Table 1, Figs 1 and 2A) was mentioned for the first time in 1871 by [1] and was investigated by [2], [3] and [4]. The site is located on the top of the La Redută Hill (La Beciuri Hill – 280-290 m a.s.l.), a secondary ridge detached from the Palanca Hill (330 m a.s.l.) by Lupăriei stream to the West and Herţa stream to the North and North-East, in the perimeter of Fundu Herţii village.

The hillfort is located on the flat plateau which has a gentle slope from NW to SE and consists of three transversal walls (numbered 1 to 3 from north to south in Fig 2A) with ridges (7-10 m in width and 0.8 to 2.2 m in height) and a lateral wall which close the settlement on all directions (5.5 m in width and 1 m in height), excepting for the North-Eastern part where recent landslides (Fig 2A) destroyed it [3]. At the base of the plateau, whose edges are landslide scarps, there is an external trench (had 3.5 to 4.5 m in width and 2.25 to 2.75 in depth) which is continuous on the North-western and Western part, in the North-eastern part being destroyed by the landslides [3]. The trench continues in the Eastern part of the plateau. Besides the trench, at various distances from the plateau (100-300 m), there are remains of an external continuous palisade built on top of a wall, which had 1 to 4.5 m in width and 0.25 to 1 m in height [3].

The walls are built from earth and were plated by stones to the external trench and by wood in the internal part. The defensive system was completed by wood palisades [3]. The hillfort was built in the 1050-950 yr. BP period (transversal walls 1 and 2, longitudinal wall, external trench, and palisades) but was destroyed at a particular time, being rebuilt, with the addition of a third transversal wall in the 950-750 yr. BP time (in which period, the external palisade and trench were probably not used anymore) [3].

The hillfort was built on the plateau after the forest was cut and burnt, tree roots, and ash being found at the base of the archaeological deposits [3]. The medieval diggings often affected Cucutenian houses platforms, their material being used for the wall construction.

**Site 11: Scutari – La Gheţărie**

The Scutari – La Gheţărie site (S11, Table 1, Figs 1, and 2B) was discovered and investigated in 1967-1970 and described in 1971 by [5]. Some surface investigations were performed showing the presence of a settlement (with a surface of 5 ha), which spans from the Podriga floodplain to the adjacent landslide right hillslope opposite to the Scutari village (Fig 2B). The archaeological rescue investigation required by dam building revealed a house and a hole where Cucuteni A, A-B, B, and C remains being found [5]. On the adjacent right hillslope, inside the perimeter of a landslide, footprints of approx. Twenty houses (Fig 2C) were found by [5] in the fresh tillage soil (can also be seen on Google Earth and aerial images of the area) on a surface of approx. 10 ha.

**Site 12: Dersca**

The Dersca – La Pisc (S12a, Table 1, Figs 1 and 2C) and Berezna (S12b, Table 1, Figs 1 and 2C) sites are located on top of two adjacent hills, Piscului Hill and Berezna Hill, in the upper part of the Buhai river catchment, a tributary of the Jijia River, North of the Dersca village. In this area, the Southern hillslope of the Rusului Hill (409.4 m a.s.l.) is incised by the Buhai Valley and two left tributaries, Buhăiaşul and Potici.

Between the Buhăiaşul and Potici streams, on the terminal slope of the Piscului Hill (338.5 m a.s.l.), La Pisc site was discovered in 1971 [6]. La Pisc hillfort was successively investigated by [6,7]. The site is located on the terminal gentle sloping part of Piscului Hill and consists of three transversal walls (noted I to III from south to north in the order of their construction - Fig 2C) with their adjacent trenches and a smaller longitudinal wall that close the hillfort toward East and South-East, joining the head of the third wall (III) [6]. A similar wall probably existed on the North-Eastern part, but here recent landslides (Fig 2C) destroy it together with a part of the terminations of the three transversal walls [6].

The first wall (I) had 44 m in length, 2.60 m in height, and a trench with 3.85 m in depth. This wall has best preserved its morphology, but nowadays, it is cut by landslide scarps at its northeastern edge (Fig 2C). The wall was covered by burnt earth (which is impermeable) and by sandstone to the external part in order to minimize erosion [6].

The second wall (II) had 84 m in length, a base of 10 m and a height of 1.25 m [6], being smoothed in the present-day topography. Its trench is partially filled and had an initial depth of 3.75 m [6]. In the trench, there are pieces of the sandstones used to cover the outer part of the wall [6].

The third wall (III) had 129 m in length, a base of 6 m [6], and is very smooth (only 0.30 – 0.40 in height) in the present-day topography. Its adjacent trench is filled and had an initial depth of 0.50 – 0.75 m and widths of 2 to 3.5 m [6]. The wall has a tetrahedral nucleus of wood with gunning earth inside [6]. The outer slope was probably covered by reddish stones that were found in the trench. The trench had two sets of wood palisades, arranged in a vertical and oblique system (45 degrees), as can be seen in other Eastern European early medieval hillforts [3,8]. The lateral wall had a length of 360 m and a height of 0.75 to 0.90 m [6].

The wall structure indicates two stages of construction [6]. The first stage walls and palisades were burnt, which gave a reddish color to the sandstones used to stabilize the outer part of the walls, and part of their material filled the trenches. Later, the wall was reconstructed using material from the trenches fill [6].

Between the Buhai and Buhăiaşul streams on the terminal slope of the Berezna Hill (345 m a.s.l.), there is a well preserved wall-trench system: the wall has 31.5 m in width and 4.5 m in height, the trench has 28.5 m in width and 4.7 m in depth; between the wall and the trench the distance is 26.7 m. The site was first mentioned by [1] but has not been thoroughly investigated archaeologically, being considered of Hallstatt age (3150-2650 yr. BP) (http://ran.cimec.ro/sel.asp?descript=dersca-dersca-botosani-asezarea-hallstatt-de-la-dersca-berezna-cod-sit-ran-37020.01; [9]).

**Site 13: Corlăţeni – Movila Cetăţii**

The Corlăţeni – Dealul Cetăţii site (S13, Table 1, Figs 1 and 2D) is located east of the Corlăţeni village, where the lower part of the Southern hillslope of the Cetatea Hill (201.36 m a.s.l.) is covered by a settlement with continuous population from Neolithic to 1650-1550 yr. BP [3,10,11]. On the ridge, there are a medieval fortified settlement (Corlăţeni – Dealul Cetăţii, first mentioned by [1] and investigated by [11]) and Yamnaya tumuli mounds [10,11]. The archaeological sites from around the Corlăţeni – Dealul Cetăţii site were named in Fig 2D according to their Romanian nomenclature from the Romanian Archaeological Repertoire (RAN-CIMEC, http://ran.cimec.ro/). One of these tumuli is located on the edge of the ridge (Movila din Dealul Cetăţii de la Corlăţeni), a quarter of its surface being affected by landslides (Fig 2), which generated an 8 m scarp in which the geological clays occurs. The shape of the tumulus is ellipsoidal, 50 m in diameter and 2 m in height, but the present-day situation represents the erosional and depositional result of a higher and smaller initial mound (Fig 2). This initial mound was smoothed by erosion, and the eroded material was deposited around its initial margins [11]. The age of the mound is Bronze Age (Yamnaya Culture) [11].

**Site 14: Plugari – Movila Balş**

The Movila Balş site is located 2.5 km north-east of Plugari village (S14, Table 1, Figs 1 and 3B) on top of the Balş Hill (180.8 m a.s.l.). The Balş Hill is a cuesta with the western hillslope as a scarp, steep and short (80 m local relief and 500 m length), and the eastern hillslope as a dipslope, gentle sloped and long (95 m local relief and 1.2 km in length). The Coroleuca stream to the west and Recea stream to the east (both tributaries on the right side of the Miletin River) delineate the hill. The tumulus mound has a slight ellipsoidal shape with a small axis of 49.6 m, the big axis of 55.8 m, and a height of 5.3 m. Its northwestern part is affected by a landslide scarp. Considering the mound’s shape and location, the same age as for site 13 was assigned to this site.

**Site 15: Prăjeni – Movila Robului**

The Odăii Mound site is located 2.6 km southeast of Prăjeni village, east of Lupăria village (S15, Table 1, Figs 1 and 3C) on the western hillslope of Ţiglău Hill (185.23 m a.s.l.) at the base of the hillslope (locally called Coasta Odăii), close to the right bank of Robul stream (which is incised 6 m from the base of the hillslope). The mound was built over a relict landslide deposit (Fig 3C), which is 4 m higher than the Robul floodplain. The tumulus mound has a slight ellipsoidal shape with asmall axis of 48.7 m, the big axis of 53.7 m, and a height of 2.4 m. Considering the mound’s shape and location, the same age as for site 13 was assigned to this site.

**Site 16: Coarnele Caprei – Movila Boului**

The Movila Boului site (S16, Table 1, Figs 1 and 3D) is located 8.3 km northwest of Coarnele Caprei village and 4.2 km North-West of Petroşica hamlet on the top of the Boului Hill (175.2 m a.s.l.). The Boului Hill is a symmetric ridge, delineated by Contoş (Gurguiata) river to the west (98 m local relief) and Minciunei stream to the east (58 m local relief). The relative relief toward the east is 50 m, and toward the west is 75 m. The tumulus mound has a slight ellipsoidal shape with a small axis of 57.3 m, the big axis of 64.1 m, and a height of 2.8 m. Its eastern part is affected by a landslide scarp (Fig 3D). Considering the mound’s shape and location, the same age as for site 13 was assigned to this site.

**Site 17: Todireşti – La Şanţuri**

Todireşti – La Şanţuri site (S17, Table 1, Figs 1 and 3A) is located at 2.6 km northwest from Vânători village (Todireşti commune) on a secondary ridge called Şanţurilor Hill (533.5 m a.s.l.) which is detached toward south from the main hill called Podul de Lut Hill (534 m a.s.l.) by the Urlea sub Potcoavă stream. This flat and 1 km long secondary ridge is very narrow (50 to 180 m) due to intense landsliding on both its western (left side of Vladnic stream valley) and eastern (right side of Urlea sub Potcoavă stream valley) hillslopes.

The geto-dacian hillfort located on top of this secondary ridge is destroyed by retrogressive landslides, only three short wall and trench systems having survived (Fig 3A). From north to south, these wall and trench systems have 100, 30, and 40 m in length. The northern system has two segments, which connect in a 120° angle in an area where an entry was placed. The northern trench has a depth of 1.1 m, and the adjacent wall has a height of 1.1 m. The central trench has a depth of 1.2 m, and the central wall has a height of 1.3 m. The southern wall and trench system is composed of four parallel ditches with depths of 1.4, 1 and 0.5 m, extending from the interior of the fortification towards the exterior, separated by three ridges, from which only the interior one, with a height of 1.4 m, is similar to a wall.

The site has not been thoroughly investigated archaeologically but remains from the Cucuteni A, and the geto-dacian La Tène cultures were found inside its perimeter (http://ran.cimec.ro/sel.asp?descript=todiresti-todiresti-iasi-situl-arheologic-de-la-todiresti-la-santuri-cod-sit-ran-99548.01).

**Site 18: Cotnari - Horodiştea**

The Horodiştea site (S18, Table 1, Figs 1 and 4A) is located inside the boundary of the Horodiștea village (Cotnari commune, Iași County) and represents an adjacent extension of the Cătălina hillfort [13-16]. The wall and the trench system have a length of 2 km and is protecting the Western flank of the Cătălina hillfort stretching from the Western hillslope of Cătălina Hill (394 m a.s.l.) to the Pietros Hill (280 m a.s.l.). On the northern part, its topography is smoothed by the land reclamation works (terracing). In the southern part, where the initial topography is still visible, the wall has up to 4.5 m in height and up to 24.5 m in width while the trench has up to 5 m in depth, and it is up to 28 m wide. On the southern part of the hillfort, there is another small wall (150 m in length), 0.5 m in height that bounds a landslide scarp. With our LiDAR and field reconnaissance mapping (Fig 4A), we did not find the full extent of the defensive system mapped by [16] on aerial imagery (especially the eastern extension of the hillfort, where the shading given by the aerial imagery corresponds with a leveled landslide flank).

**Site 19: Filiaşi – Dealul Mare-Boghiului**

The Filiași – Dealul Mare (Boghiului) site (S19a, Table 1, Figs 1 and 4B) is located south of Filiași village on a secondary ridge (185.72 m a.s.l.), detached from the main ridge of Boghiului Hill (195 m a.s.l.). The relief of the secondary ridge is of 90 m, relative to the local Oaia floodplain (89 m a.s.l.) the top of the ridge having an NW to SE orientation and a rounded shape/profile.

The site was investigated in 1931 [17], 1955 [18] and 1984-1986 [19] through archaeological diggings, which revealed a Cucuteni A3 settlement, and by [20] who through geophysical investigation methods, found an inner settlement with a significant density of houses disposed in a semicircular pattern, protected by double trenches, and an outer settlement, with a smaller density and protected by a set of three parallel curvilinear ditches (Fig 4B). Many of the positive anomalies identified by [20] using the LiDAR DEM and matched by positive anomalies on the magnetic survey results, actually corresponds to the micro-topography created by the army during the World War II (trenches from the Northern part of the Chalcolithic settlement), by agrotechnical measures (the small ridge around the cultivated fields) or by clay extraction (the North-Eastern part of the main settlement, south of the army trenches).

On top of Boghiului Hill, at 190 m a.s.l., there is a tumulus (S19b, Table 1, Figs 1, 4D and 7J,K,M), which is not archaeologically investigated. Considering the mound’s shape and location, the same age as for site 13 was assigned to this site.

**Site 20: Hăbăşeşti – La Silişte**

The Hăbăşeşti – La Silişte site (S20, Table 1, Figs 1 and 4C) is located in the North-East of Hăbășești village on a rectangular plateau at the edge of the Strunga Hill (295.8 m a.s.l.). The incision of the Upper Strunga river reaches generated the landslides on the amphitheater-like morphology (Fig 8I) of the southeastern hillslope of Strunga Hill and the northern hillslope of Hăbășești Hill (297.4 m a.s.l.). The rectangular area on which the Chalcolithic settlement is located is 150 m long and 100 m wide, oriented NW to SE (Fig 8J), and is bordered by steep landslide scarps on the northern, eastern and southern edges (Fig 8J,K,E). The western part is connected to the hilltop through a saddle (Fig 8I). In this area, the Cucutenian settlement was protected by two trenches, the exterior one having 105 m in length, 7.1 m in width and 3.15 m in depth, and the interior one 103 m in length, 6 m in width and 2.30 m in depth [21]. The eastern edge of the promontory was affected by landslides in 1930-1932, the old scarp sliding down and creating two slices around 4-5 m lower than the initial ridge level [21]. The top slice has two in situ Cucutenian houses, which lead [21] to affirm that this slided area is older than the Cucutenian settlement because the houses from this area are not disturbed. If we analyze the geometry of the two slices (which is typical for reversed slopes), we can affirm that both of them have the same age because they are similar in shape and vertical displacement (Fig 8I). By chance, the fracture of the scarp appeared exactly between two Cucutenian houses, and the displacement was almost vertical, so the houses from this slice are not disturbed. In the settlement, 44 houses, and more than 80 food storage pits were discovered [21].

In the site there were found remains from the Hallstatt, Daco-Roman, and Medieval periods (<http://ran.cimec.ro/sel.asp?descript=habasesti-strunga-iasi-situl-arheologic-de-la-habasesti-la-siliste-cod-sit-ran-99281.01>).

**Site 21: Pocreaca – Punct Cetăţuia**

The Pocreaca –Cetăţuia site (S21, Table 1, Figs 1 and 5C) is located on a promontory detached from the Cetăţuia (Nemţoaica) Hill (298.6 m a.s.l.) between Slobozia (Pocrecuţa) stream (right affluent of Vasluieţ River) and its left tributary, Nemţoaica stream. The 350 m long and 80 m wide ridge is oriented from NW to SE and at its terminal part, has three walls separated by three trenches (noted from 1 to 3, from south to north in Fig 5C). The wall and the trench systems are profoundly affected by recent landslides scarps (Fig 5C). [22] investigated the site and discovered that, besides the triple wall-trench system, the Hallstatt site was enclosed toward the gentle northwest part by a double arcuate wall (Fig 5C). From the archaeological remains [22] estimated the following dimensions of the wall-trench systems: (i) wall 1 is the highest (1.7 m) being 20 m long and 19 m wide, its remains filling up almost entirely trench 1 (Fig 8C); (ii) wall 2 has the biggest length (45 m) but is only 17 m wide and 1.3 m high, its trench having 30 m in length, 17 m in width and 2.7 m in depth; (iii) wall 3 has only 9.5 m in length and is very smoothed; its trench having 45 m in length 16 m in width and 2.3 m in depth; (iv) the arcuate walls have 2 m in width and only 30-40 cm in height, the northeastern having 120 m in length and the northwestern 80 m. [22] argues that the fortress was built and occupied between 3050 and 2750 yr. BP. Before this period, the site was occupied by a Cucuteni culture settlement, which was disturbed by the thraco-getic fortress.

**Site 22: Creţeşti – Dealul Cetăţii**

The Creţeşti – Dealul Cetăţii site (S22, Table 1, Figs 1 and 5A) is located 2 km north of Creţeştii de Sus village and at the northwestern edge of the Movila Cetăţuia Hill (353.6 m a.s.l.), on a secondary ridge between Vlăscineasa stream and one of its left tributary streams. The hillfort consists of a wall and a trench of semicircular shape at the upper part of the ridge [24], where is the contact with the main hill ridge, and a terrace which is barely visible in the topography and which surrounds the ridge nose. This terrace is similar to the terrace present on the southern part of Moşna hillfort [14,23], where it was built at the upper part of a landslide scarp. At the northern edge of the hillfort, an old landslide scarp has cut this terrace (Fig 5A).

The wall has 300 m in length, 4.5 – 5 m in height, and a base of 15 to 20 m. [24] mention the presence of an eastern wall with a base of 15 m and a height of 2-3 m, but on LiDAR data is not visible such a feature. The trench has a circular shape with the biggest width measuring 15 m and a depth of 2-3 m. The archaeological complex has a surface of 5-7 ha, and it is considered to be from 2350-2250 yr. BP [24].

**Site 23: Corni-Albeşti –Vladnic**

The Vladnic site (S23, Table 1, Figs 1 and 5B) is located 7 km north of Vutcani village and 3 km east of Corni village. The location of the small fortified enclosure (with an area of 1.5 ha) is placed at the base of the northwestern hillslope of Mălăişte Hill (315.2 m a.s.l.). Between Mălăişte Hill and Ursoii Hill (317.3 m a.s.l.), the Cetăţuii stream (a left tributary of Idrici River), has incised a V-shaped valley, its steep right bank giving the protection for the flat base of the hillslope where the fortified enclosure is located. The wall and trench system were built toward the north, east, and south-east to block the access from the gently sloped area of the hillslope. The wall has a length of 140 m, a height of 4 – 4.5 m, and a base of 15 – 18 m. The trench has a width of 15 – 18 m and a depth of 1.5 to 3 m. At the eastern part of the fortified enclosure, the wall-trench is more like a terrace, situation which is similar to the steep southern part of the Moşna hillfort [23]. At the southern and western edges, the wall and trench are destroyed by old and recent landslides triggered by the river down-cutting (Fig 5B). The archaeological complex has an area of 0.8 ha and is considered to be from 2350-2250 yr. BP [24].

**References**

1. Odobescu Al, [Antiques remains from Dorohoi County]. In: Opere Complete, vol. 3, 1908; p. 118-166. Romanian.
2. Verona P, [Memory related to discoveries made in the Herții region of Dorohoi County]. Revista de Istorie Română. 1936; 5-6: 633–637. Romanian.
3. Petrescu-Dîmboviţa M, Teodor DG. [Fortification systems from Early Medieval Period at the Eastern Carpathians. The settlement from Fundu Herţii – Botoşani County]. Iasi: Junimea Press; 1987. Romanian.
4. Ambrojevici C, Popovici R, Zur vorgeschichtlichen und mittelalterlichen Vergangenheit des Bezirkes Dorohoi. Dacia. 1945; 9-10: 115–125. German.
5. Crîşmaru A. [New archaeological discoveries on Podriga Valley - Botoşani County]. Hierasus. 1979; 2: 97–120. Romanian.
6. Teodor DG, [The early medieval stronghold from Dersca – Botoșani]. Hierasus. 2001; 11: 107–130. Romanian.
7. Teodor DG, Les établissements fortifiés des régions est-Carpatiques de la Roumanie aux VIIIe-XIe siècles de notre ère. Slovenska Archeologia. 1978; 26(1): 69–77. French.
8. Kudrnáč J, [Archaeological research at Klučov, Czechoslovakia]. Studii și cercetări de istorie veche. 1967; 18(2): 269–281. Romanian.
9. Florescu AC, Florescu M. 2012. [The thraco-getic fortresses from Stâncești (Botoșani County)]. Suceava: Cetatea de Scaun Press; 2012. Romanian.
10. Nestor I, Alexandrescu A, Brătianu A, Comşa E, Perju S, Vieru I. [The reports of archaeological collectives concerning the diggings from 1949 campaign – Study of the society from Early Barbarian Age from Northern Moldavia – Activity on Iaşi-Botoşani-Dorohoi archaeological sites]. Studii și cercetări de istorie veche. 1950; 1: 27–32. Romanian.
11. Nestor I, Alexandrescu A, Comşa E, Zaharia-Petrescu E, Zirra V. [The diggings from Jijia Valley archaeological site - Iaşi-Botoşani-Dorohoi - in 1950]. Studii și cercetări de istorie veche. 1951; 2: 51-76. Romanian.
12. Petrescu-Dîmboviţa M, Bîrsan M, Bold E, Boroneanț V, Cazacu P, Dinu M, et al. [The Hlincea Iași archaeological site]. Studii și cercetări de istorie veche. 1952; 4(1-2): 233-251. Romanian.
13. Zaharia N, Petrescu-Dîmbovița M, Zaharia E, 1970. [Settlements from Moldavia: from Paleolithic to the 18th Century]. Bucharest: Romanian Academy Press. Romanian.
14. Florescu AC, [Some considerations regarding the thraco-getic fortresses (Hallstatt) from the first millennium before our era from the territory of Moldavia]. Cercetări Istorice. 1971; 2:103–118. Romanian.
15. Florescu AC, [New aspects on thraco-geto-dacic fortifications from the second half of the first millennia BC, discovered in Moldavia]. Revista Monumentelor şi Muzeelor – Monumente Istorice şi de Artă. 1980; 49(1): 11–18. Romanian.
16. Ștefan AS. Les fortifications du premier Age du Fer de Cotnari (Départment de Iassy, Moldavie, Roumanie). Photo-Interprétation. 1990; 29(6): 45-57. French.
17. Tafrali O, [The prehistoric resort of Boghiu]. Arta şi Arheologia. 1937; 11-12: 51–54. Romanian.
18. Berlescu N, [Cucutenian settlements from Războieni and Prigorenii Mici - Tg. Frumos county, Iași Region]. Studii și Cercetări Științifice – Iași. 1955; 6(3-4): 151–163. Romanian.
19. Boghian D, [The cucutenian communities from Bahlui catchment]. Suceava: Stefan cel Mare University of Suceava University Press. Romanian.
20. Asăndulesei A, Inside a Cucuteni settlement: remote sensing techniques for documenting an unexplored eneolithic site from Northeastern Romania. Remote Sensing. 2017; 9: 41.
21. Petrescu-Dîmboviţa M, Brăteanu A, Dincă M, Dinu M, Florescu A, Ordentlich I, et al. [Trușești archaeological site]. Studii și cercetări de istorie veche. 1954; 5(1-2): 8-28.
22. Iconomu C. [Archaeological research in the Hallstat fortress from Pocreaca – Iaşi]. Arheologia Moldovei. 1996; 19: 21–56. Romanian.
23. Niculiță M, Mărgărint MC, Santangelo M. Archaeological evidence for Holocene landslide activity in the Eastern Carpathian lowland. Quaternary International. 2016; 415: 175–189.
24. Florescu AC, Melinte G. [Hallstattian fortresses, recently discovered in northeastern part of Central Moldavia]. Carpica. 1971; 4: 129–132. Romanian.
